# «Salivaomics» of Different Molecular Biological Subtypes of Breast Cancer

**DOI:** 10.3390/cimb44070211

**Published:** 2022-07-05

**Authors:** Lyudmila V. Bel’skaya, Elena A. Sarf

**Affiliations:** Biochemistry Research Laboratory, Omsk State Pedagogical University, 644043 Omsk, Russia; nemcha@mail.ru

**Keywords:** salivaomics, breast cancer, biomarkers, saliva, molecular biological subtype, HER2 status, estrogen receptors, progesterone receptors

## Abstract

The aim of the study was to determine the metabolic characteristics of saliva depending on the molecular biological subtype of breast cancer, as well as depending on the expression levels of HER2, estrogen receptors (ER), and progesterone receptors (PR). The study included 487 patients with morphologically verified breast cancer and 298 volunteers without breast pathologies. Saliva samples were obtained from all patients strictly before the start of treatment and the values of 42 biochemical indicators were determined. It has been established that the saliva of healthy volunteers and patients with various molecular biological subtypes of breast cancer differs in 12 biochemical indicators: concentrations of protein, urea, nitric oxide, malondialdehyde, total amino acid content, and activity of lactate dehydrogenase, alkaline phosphatase, gamma-glutamyltransferase, catalase, amylase, superoxide dismutase, and peroxidases. The saliva composition of patients with basal-like breast cancer differs from other subtypes in terms of the maximum number of indicators. Changes in biochemical indicators indicated an increase in the processes of lipid peroxidation and endogenous intoxication and a weakening of antioxidant protection, which correlates with the severity of the disease and the least favorable prognosis for this subtype of breast cancer. An analysis was made of the individual contribution of the expression level of HER2, estrogen, and progesterone receptors to changes in the biochemical composition of saliva. The HER2 (−)/HER2 (+) group, which should be considered as a single group, as well as ER-positive breast cancer, differ statistically significantly from the control group. For ER/PR-positive breast cancer, a more favorable ratio of saliva biochemical indicators was also noted compared to ER/PR-negative breast cancer.

## 1. Introduction

Breast cancer is the most common female cancer worldwide [1,2,3,4]. Despite the improvement in early diagnosis and the active use of adjuvant drug treatment, only 59% of patients in Russia survive the 5-year follow-up period [5], and mortality from breast cancer in Russia does not decrease due to late detection of the disease [6,7]. The proportion of early breast cancer (cancer in situ and stage I) is critically small: the proportion of non-invasive breast cancer was less than 1%, and stage I breast cancer was only 18.3%, which focuses attention on the existing problem of early diagnosis of the disease [8]. Nevertheless, the current level of knowledge about the molecular mechanisms of the onset and development of breast cancer, its sensitivity or resistance to various drugs, allows the transition from averaged standard therapy regimens to the so-called “personalized medicine” [9,10], i.e., the appointment treatment in accordance with the individual characteristics of the patient and the biological characteristics of the tumor. Breast cancer is a heterogeneous disease [11]. This heterogeneity, which has been characterized at the histological level for decades, is now being assessed at the molecular genetic level, so that each type of tumor is an independent disease. The high heterogeneity of breast cancer makes its molecular characterization fundamentally important, based not only on the determination of gene mutations and gene expression profile, but also on biological markers [12]. Examples of such markers include: expression of estrogen receptors (ER) and progesterone receptors (PR), expression of the proliferation marker Ki-67 in the active phase of the cell cycle (G1, S, G2, and mitosis) and its absence in resting cells (G0), and the expression of the type 2 human epidermal growth factor receptor (HER2) are also isolated [13,14,15]. Determination of these characteristics is possible only after surgical treatment or tumor biopsy, and the results can be significantly distorted after preoperative chemotherapy and radiotherapy. For some patients, data on the molecular characteristics of the tumor cannot be obtained for a number of reasons. In this regard, it is necessary to search for alternative non-invasive markers that can characterize individual types of tumors and act as diagnostic and prognostic signs [16].

Recently, evidence has been accumulating demonstrating the diagnostic and prognostic value of saliva as a promising alternative to liquid biopsy [17,18,19,20,21,22,23]. Saliva is a complex body fluid that contains a wide range of proteins, as well as DNA, mRNA, microRNA (miRNA/miR), metabolites, and microbiota [24]. As a diagnostic approach, saliva has many biochemical advantages over blood and tissues, such as non-invasiveness, ease of storage, cost-effectiveness of collection, and dynamic availability for monitoring with less discomfort for the patient [25]. Continuous progress in saliva research has allowed the scientific community to coin the term “salivaomics” [26,27]. Changes in the genome, microbiome, epigenome, transcriptome, proteome, and metabolome of saliva can be used for diagnosis, assessment of individual risk, prognosis, and disease monitoring [27].

The literature provides data on the study of the composition of saliva in breast cancer [28,29,30,31,32,33,34,35,36,37,38,39,40,41,42,43,44]. Saliva biomarkers have been shown to achieve a sensitivity of 73% (72–74) and a specificity of 74% (72–76) in the diagnosis of breast cancer [45]. However, only one study showed the relationship between the saliva metabolome and the molecular biological subtype of breast cancer [46]. Previously, we have shown that there are changes in the metabolic profile of saliva in breast cancer [47]. It has been shown that concentration of total protein, urea, uric acid (UA), the total content of α-amino acids and lipid peroxidation products, and the activity of metabolic and antioxidant enzymes (in particular catalase) of saliva changed significantly in breast cancer. This study is one of the largest to date and includes patients with early stages of breast cancer (226/487). The metabolic features of the composition of the saliva of patients depending on the prevalence of the process and the histological type of breast cancer are considered [47]. In this work, we analyze the changes in 42 biochemical indicators of saliva depending on the molecular biological subtype of breast cancer, as well as depending on the expression levels of HER2, estrogen receptors (ER), and progesterone receptors (PR).

## 2. Materials and Methods

### 2.1. Study Design and Group Description

The study included 487 patients of the Clinical Oncological Dispensary in Omsk. The sample size of this study was the number we could recruit within the study periods (January 2015–May 2017). All patients had histologically diagnosed with breast cancer. None had received any prior treatment, including hormone therapy, chemotherapy, molecularly targeted therapy, radiotherapy, surgery, etc. The inclusion criteria that were considered include: the age of patients 30–70 years, the absence of any treatment at the time of the study, the absence of signs of active infection (including purulent processes), and good oral hygiene. The volunteers included in the study did not reveal any clinically significant concomitant diseases other than cancer pathology (in particular, diabetes mellitus, cardiovascular pathologies, etc.) that could affect the results of the study. Exclusion criteria included lack of histological verification of the diagnosis. The control group consisted of 298 healthy patients, in whom no breast pathology was detected during routine clinical examination. A detailed description of the study group is given in Table 1.

### 2.2. Determination of the Expression of the Receptors for Estrogen, Progesterone and HER2

The Allred Scoring Guideline was used to assess the expression level of estrogen receptors (ER) and progesterone (PR) (Table 1) [48]. The calculated integrative indicator allows us to define the case under study in one of four main groups: a group with an expression level of 0 points (complete absence of stained nuclei, indicated by “−”), a group with a weak color level (index from 2 to 4 points, indicated by “+”), a group with an average level of expression (index from 5 to 6 points, indicated by “++”), and a group with a high level of expression (index is from 7 to 8 points, indicated by “+++”). When determining one of the four categories of the receptors for estrogen, progesterone, and HER2 expression levels (−, +, ++, +++), the recommendations of ASCO/CAP were followed [49]. Determination of HER2 expression was carried out with immunohistochemical method, with an indeterminate result (++) used to confirm the HER2 status. Following this, a study was carried out with in situ hybridization (FISH). HER2-status assessed as “−” and “+” was considered negative, assessed as “+++” was considered positive, and assessed as “++” was assigned to an undefined level. Additionally, breast cancer sub-classification differentiates these tumors into five groups: basal-like (BL, Triple-negative), luminal A-like, luminal B-like (HER2-negative), luminal B-like (HER2-positive), and non-luminal (Table 1). The determination of the molecular biological subtype was carried out as standard with a combination of the status of HER2, estrogen, and progesterone receptors and the level of Ki67. [7].

### 2.3. Saliva Collection and Analysis

Saliva (5 mL) was collected from all participants prior to treatment. Collection of saliva samples was carried out on an empty stomach after rinsing the mouth with water in the interval of 8–10 am by spitting into sterile polypropylene tubes; the salivation rate (mL/min) was calculated. We did not find significant differences in the salivary flow rate in the studied groups, so they were not shown in the tables below. Saliva samples were centrifuged (10,000× *g* for 10 min) (CLb-16, Moscow, Russia), and the supernatant fraction was used for subsequent analysis. Biochemical analysis was immediately performed without storage and freezing using the StatFax 3300 semi-automatic biochemical analyzer (Awareness Technology, Palm City, FL, USA) [50]. A full cycle of studies was performed within 3–4 h from the moment of collection. Protease inhibitors were not used.

The pH, mineral composition (calcium, phosphorus, sodium, potassium, magnesium, chlorides), the content of urea, total protein, albumin, uric acid, α-amino acids, imidazole compounds, seromucoids, nitric oxide—NO, lactic, pyruvic, and sialic acids, as well as the activity of enzymes (aminotransferases—ALT, AST; alkaline phosphatase—ALP; lactate dehydrogenase—LDH; gamma-glutamyl transpeptidase—GGT; α-amylase), were determined in all samples. The content of substrates for lipid peroxidation processes (diene conjugates—DC, triene conjugates—TC, Schiff bases—SB, malondialdehyde—MDA) and indicators of endogenous intoxication (MM—middle molecules) were determined. We determined the MM at wavelengths of 254 and 280 nm; they are designated MM 254 and MM 280, respectively [51]. Additionally, we assessed the activity of antioxidant enzymes (catalase—CAT, superoxide dismutase—SOD, antioxidant activity, peroxidase). The potential value of calculating a number of ratios has been previously shown, for example, Na/K, Ca/P, AST/ALT, SOD/Catalase, SOD/Peroxidase, SB/(DC+TC), SB/TC, and MM 280/254. In addition to the direct evaluation of 34 biochemical salivary indicators, we additionally evaluated the values of 8 ratios, so the total number of indicators was 42.

### 2.4. Statistical Analysis

Statistical analysis was performed using Statistica 13.3 EN software (StatSoft, Tulsa, OK, USA); R version 3.6.3; RStudio Version 1.2.5033; FactoMineR version 2.3. (RStudio, version 3.2.3, Boston, MA, USA) with a nonparametric method using the Mann–Whitney U-test and the Kruskal–Wallis H-test. The description of the sample was made by calculating the median (Me) and the interquartile range as the 25th and 75th percentiles [LQ; UQ]. Differences were considered statistically significant at *p* > 0.05.

A principal component analysis (PCA) was performed using the PCA program in R. The choice of variables for the PCA method was carried out according to the results of comparison of biochemical indicators in the studied groups. When comparing two groups, we used the Mann–Whitney test; when comparing three groups or more, we used the Kruskal–Wallis test. Next, we selected indicators for which the differences between all groups are significant at the *p* < 0.10 level. PCA results were presented in the form of factor planes and corresponding correlation circles. In each case, the figures show only the first two principal components (PC1 and PC2). The color of the arrows on the correlation circle changed from blue (weak correlation) to red (strong correlation) as shown on the color bar. The orientation of the arrows characterized positive and negative correlations (for the first principal component, we analyzed the location of the arrows relative to the vertical axis; for the second principal component, relative to the horizontal axis). The significance of the correlation was determined by the correlation coefficient (r): strong-r = ± 0.700 to ± 1.00, medium-r = ± 0.300 to ± 0.699, weak-r = 0.00 to ± 0.299.

## 3. Results

### 3.1. Changes in the Biochemical Composition of the Saliva of Patients with Breast Cancer, Depending on Its Molecular Biological Subtype

**Figure 1:**
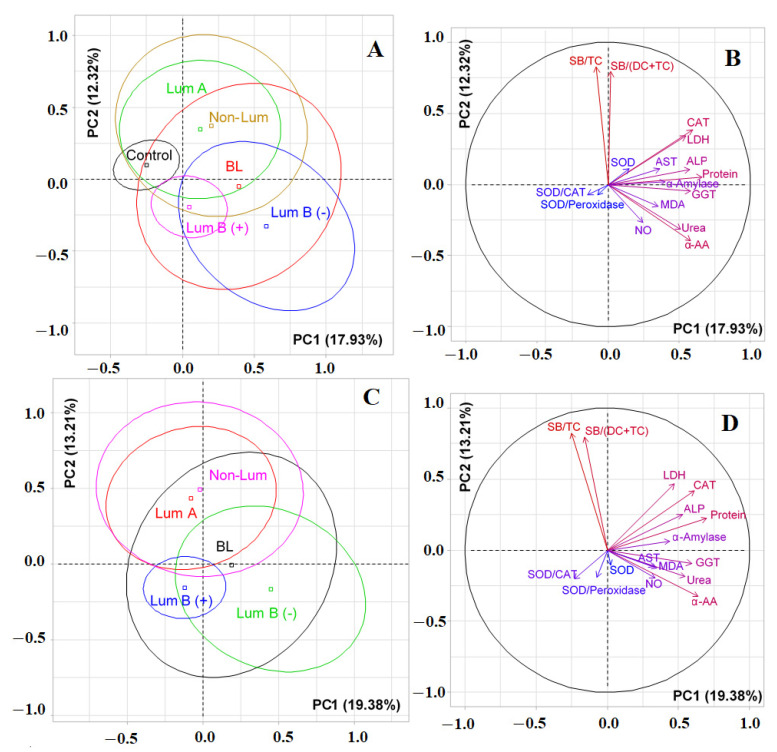
Individuals factor map (PCA) with control group (**A**) and without control group (**C**); variables factor map with control group (**B**) and without control group (**D**). LDH—lactate dehydrogenase, CAT—catalase, ALP—alkali phosphatase, AST—aspartate aminotransferase, MDA—malondialdehyde, GGT—gamma glutamyltransferase, α-AA—α-Amino acids, SB—Schiff Bases, TC—triene conjugates, DC—diene conjugates, SOD—superoxide dismutase.

At the first stage of the study, it was shown that the biochemical composition of saliva in different molecular biological subtypes of breast cancer had differences (Supplementary Tables S1 and S2). The values of biochemical indicators of saliva in the control group, as well as in various molecular biological subtypes of breast cancer, are given in Supplementary Tables S1 and S2. Table 2 below shows the deviation values of the average content of each indicator of saliva from the corresponding values for the control group. We have identified 12 biochemical indicators for which the differences between the groups are statistically significant (Table 2). Selected biochemical indicators were used to compare groups by PCA analysis (Figure 1).

It was shown by PCA analysis that there was no complete separation of all the studied groups (Figure 1). The first principal component (PC1) separated the control group (to the left of the vertical axis) and all groups of patients with breast cancer (to the right of the vertical axis) (Figure 1A). The maximum contribution to the separation was made by protein (*r = 0.6587*), the total content of α-amino acids (*r = 0.5804*) and urea (*r = 0.5088*), as well as the activities of catalase (*r = 0.5933*), GGT (*r = 0.5756*), ALP (*r =0.5732*), LDH (*r = 0.5451*), and α-amylase (*r = 0.4008*). The separation by the first principal component was statistically significant (*p = 0.0052*). The division of groups relative to the horizontal axis was due to the contribution of lipid peroxidation indicators, high correlation coefficients were determined for the SB/TC-ratio and SB/(DC+TC)-ratio and amounted to 0.8241 and 0.7935, respectively (Figure 1B). At the same time, the groups of luminal A and non-luminal breast cancer, as well as both subgroups of luminal B breast cancer, turned out to be close to each other (Figure 1A). If we compare only breast cancer patients with each other, then the trend persisted (Figure 1C). Subgroups of luminal A and non-luminal breast cancer were distinguished on the diagram by a single field (Figure 1C). The vertical axis made it possible to distinguish groups of luminal B (−) and basal-like breast cancer (to the right of the axis) from the rest (Figure 1D). The contribution to the separation was made by the same parameters as when taking into account the control group (Figure 1D); however, in this case, the separation was not statistically significant. The horizontal axis also divided luminal A and B breast cancers (Figure 1C). The division was characterized by lipid peroxidation indices and was statistically significant (*p* = 0.0083).

**Figure 2:**
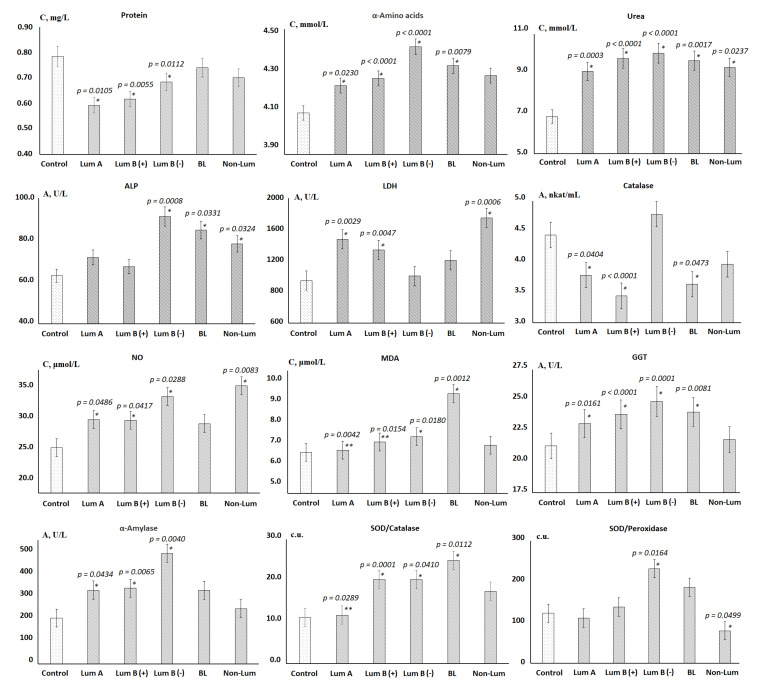
Biochemical composition of saliva depending on the molecular biological subtype of breast cancer. Differences between groups were calculated using the Wilcoxon matched pairs test with the Bonferoni correction at *p* < 0.05; *—differences with the control group are statistically significant, **—differences with BL are statistically significant. C—concentration, A—activity.

The values of biochemical indicators, which significantly change in the studied groups, are shown in Figure 2.

It was shown that the total protein content decreased in all groups, however, it was statistically significant only for the luminal subtypes. Against the background of a decrease in protein content, the content of α-amino acids and urea increased. The activity of enzymes changed ambiguously, so the subgroups of luminal A and B (+) breast cancer are similar in the nature of changes in the activity of enzymes (Figure 2). These subgroups were characterized by a slight increase in the activity of ALP, an increase in the activity of LDH and GGT, as well as a sharp decrease in the activity of catalase. For luminal B (−) breast cancer, ALP and GGT activities reached maximum values, while LDH and catalase activities remained practically unchanged. For all luminal subtypes of breast cancer, a statistically significant increase in α-amylase activity was shown. For all subgroups except for non-luminal breast cancer, an increase in the SOD/Catalase-ratio was shown, while for the non-luminal subgroup, a decrease in the SOD/Peroxidase-ratio was statistically significant (Figure 2). For basal-like cancer, the highest content of toxic products of lipid peroxidation, in particular MDA, was noted against the background of minimal catalase activity.

### 3.2. Changes in the Salivary Biochemical Composition of Breast Cancer Patients Depending on the HER2 Status

At the next stage, we tried to figure out which parameter determined the differences between the identified molecular biological subtypes of breast cancer. Table 3 shows the values of the Kruskal–Wallis criterion when separating groups according to the level of expression of HER2, estrogen, and progesterone receptors. We identified biochemical indicators whose differences between subgroups were significant at the level of 0.05 and 0.10 (Table 3). These parameters were subsequently used to compare groups with PCA analysis. A complete list of salivary biochemical indicator values for each of the subgroups is given in Supplementary Tables S3–S8.

**Figure 3:**
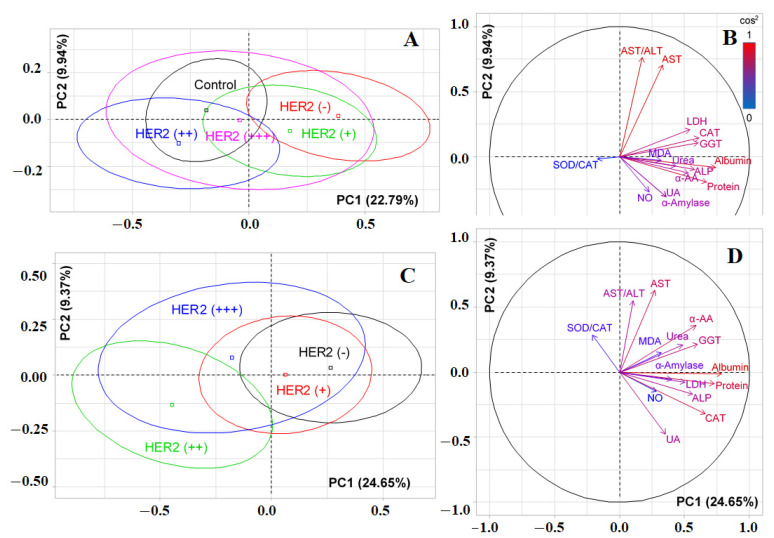
Individual factor map (PCA) with control group (**A**) and without control group (**C**); variables factor map with control group (**B**) and without control group (**D**). UA—uric acid, LDH—lactate dehydrogenase, CAT—catalase, ALP—alkali phosphatase, ALT—alanine aminotransferase, AST—aspartate aminotransferase, MDA—malondialdehyde, GGT—gamma glutamyltransferase, α-AA—α-Amino acids, SOD—superoxide dismutase.

Figure 3A shows that significant differences were observed only between the control group and HER2-negative breast cancer (*p = 0.0178*). The separation was due to the contribution of albumin (*r = 0.7412*), total protein (*r = 0.6717*), catalase (*r = 0.6123*), GGT (*r = 0.6058*), ALP (*r = 0.5767*), LDH (*r = 0.5409*), α-amino acids (*r = 0.5314*), and urea (*r = 0.4334*) (Figure 3B). The horizontal axis separated HER2-positive and HER2-negative breast cancer; however, the differences between the groups were not statistically significant (Figure 3A). In this case, positive correlations were noted for AST/ALT-ratio (*r = 0.7608*) and AST (*r = 0.7048*), while negative correlations were noted for uric acid (*r = −0.3065*) and α-amylase (*r = −0.3076*) (Figure 3B). If we consider the division without a control group (Figure 3C), then the vertical axis allowed the formation of two subgroups: HER2 (*−*) and HER2 (+), as well as HER2 (++) and HER2 (+++) (*p = 0.0189*). The horizontal axis divided the HER2 (++) and HER2 (+++) groups. In this case, α-amylase (*r = 0.4028*) was added to the list of parameters by which separation occurs for PC1, as was catalase (*r = −0.3205*) for PC2. Meanwhile, the effect of uric acid increased (*r = −0.4758*) (Figure 3D).

**Figure 4:**
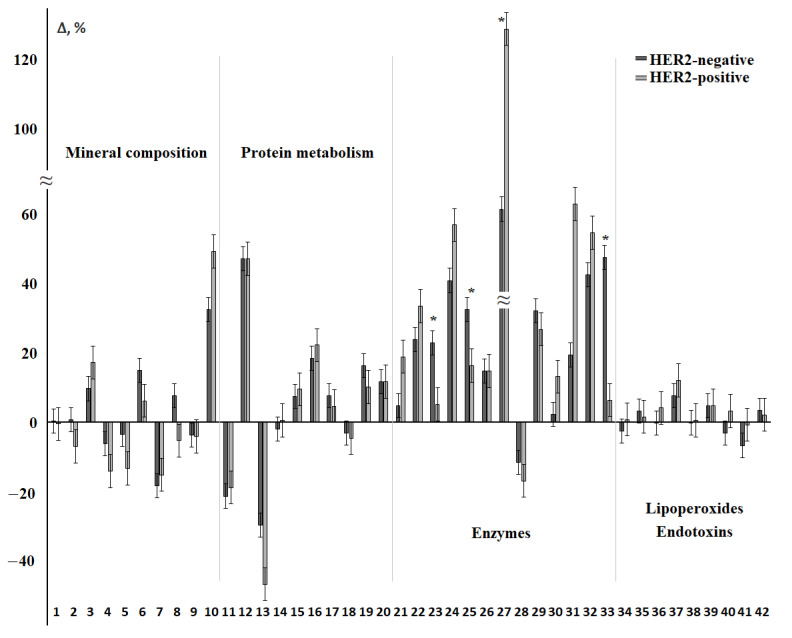
Relative change in biochemical indicators of saliva depending on the status of HER2 receptors. The interval of variation is given in comparison with the control group. The numbers of biochemical indicators correspond to the serial number in Table 2 and Table 3. *—differences between groups with HER2-positive and HER2-negative status are statistically significant, *p* < 0.05.

Figure 4 shows the relative change in each of the 42 biochemical indicators in the HER2-positive and HER2-negative breast cancer groups (Supplementary Table S3). We found that most of the indicators change in the same direction. The exception was calcium, chlorides, diene conjugates, and MM 254 nm (Figure 4). Statistically significant differences between HER2-positive and HER2-negative breast cancer were observed in AST/ALT-ratio and activity of ALP and α-amylase, as well as the SOD/Peroxidase-ratio. In general, deviations from the control group were more pronounced for HER2-positive breast cancer (Figure 4). The only indicator that statistically significantly differed between subgroups with different HER2 expression was albumin (Supplementary Table S4).

### 3.3. Changes in the Salivary Biochemical Composition of Breast Cancer Patients Depending on the ER Status

**Figure 5:**
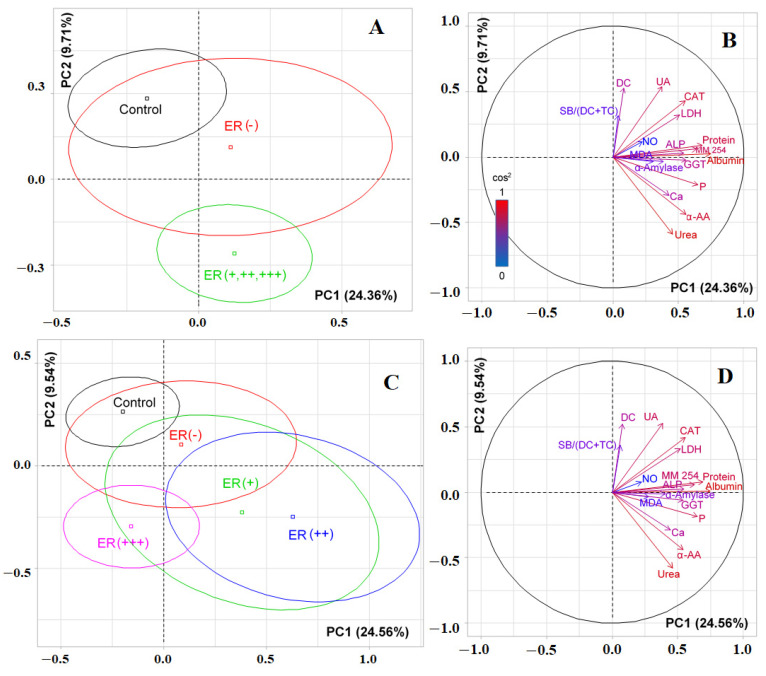
Individuals factor map (PCA) with control group (**A**) and without control group (**C**); variables factor map with control group (**B**) and without control group (**D**). UA—uric acid, LDH—lactate dehydrogenase, CAT—catalase, ALP—alkali phosphatase, MDA—malondialdehyde, SB—Schiff Bases, TC—triene conjugates, GGT—gamma glutamyltransferase, α-AA—α-Amino acids, P—phosphorus, MM—middle molecules.

According to PCA, the first principal component made it possible to distinguish the control group (to the left of the vertical axis), while the horizontal axis made it possible to distinguish ER-positive patients with breast cancer (Figure 5A). For PC1, albumin (*r = 0.7436*), protein (*r = 0.6780*), phosphorus (*r = 0.6458*), MM 254 (*r = 0.6380*), GGT (*r = 0.5587*), α-amino acids (*r = 0.5548*), catalase (*r = 0.5503*), ALP (*r = 0.5385*), LDH (*r = 0.5070*), urea (*r = 0.4554*), and calcium (*r = 0.4274*) contributed to the separation (Figure 5B). Separation by PC2 was determined by uric acid (*r = 0.5374*), diene conjugates (*r = 0.5273*), catalase (*r = 0.4276*), α-amino acids (*r = −0.4394*), and urea (*r = −0.5883*). When taking into account the degree of expression of estrogen receptors, it was shown that significant differences with the control group remained for all ER-positive subgroups, but the ER (+++) subgroup stood out separately (*p = 0.0192*, Figure 5C).

**Figure 6:**
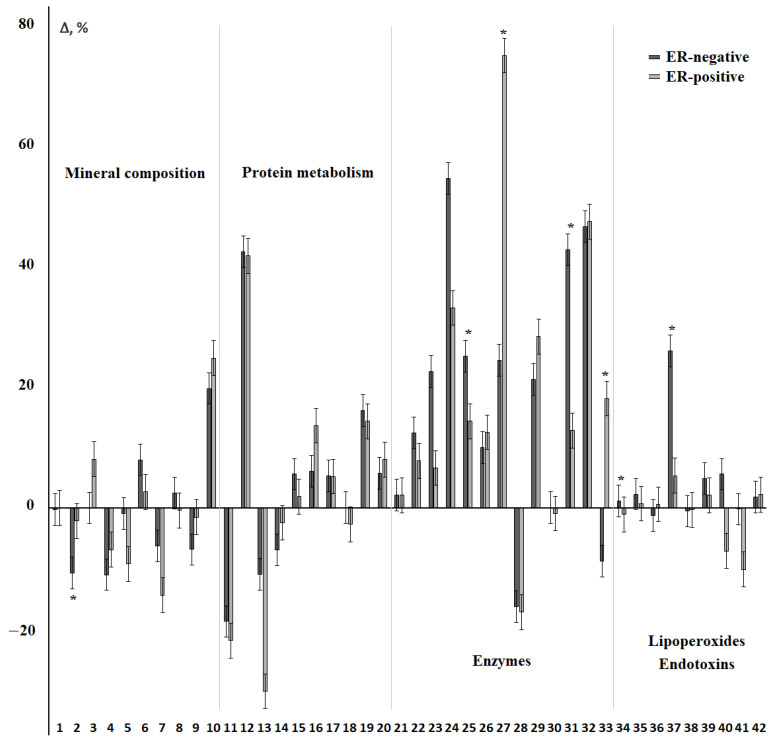
Relative change in biochemical indicators of saliva depending on the status of ER receptors. The interval of variation is given in comparison with the control group. The numbers of biochemical indicators correspond to the serial number in Table 2 and Table 3. *—differences between groups with ER-positive and ER-negative status are statistically significant, *p* < 0.05.

For ER-positive and ER-negative breast cancer, the SOD/Peroxidase ratio and the content of diene conjugates and MM 254 and 280 changed in different directions (Figure 6, Supplementary Table S5). Also statistically significant was an increase in the activity of ALP and salivary peroxidase, the level of diene conjugates, and MDA for ER-negative breast cancer. At the same time, α-amylase activity and the SOD/Peroxidase-ratio were higher for the subgroup of ER-positive breast cancer (Figure 6). When comparing subgroups with different expression of estrogen receptors, it was shown that the ER (+) and ER (+++) groups differed in the content of total protein (−31.3%, *p = 0.0002*), triene conjugates (−5.7%, *p = 0.0192*), and SB/(DC+TC)-ratio (−3.5%, *p = 0.0161*). No differences were found between the ER (+) and ER (++) groups (Supplementary Table S6).

### 3.4. Changes in the Salivary Biochemical Composition of Breast Cancer Patients Depending on the PR Status

**Figure 7:**
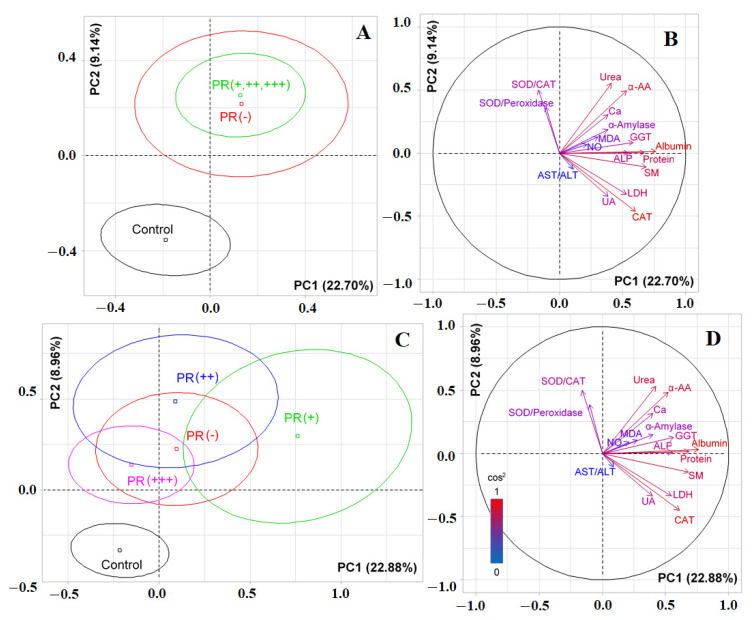
Individuals factor map (PCA) with control group (**A**) and without control group (**C**); variables factor map with control group (**B**) and without control group (**D**). UA—uric acid, LDH—lactate dehydrogenase, CAT—catalase, ALP—alkali phosphatase, ALT—alanine aminotransferase, AST—aspartate aminotransferase, MDA—malondialdehyde, GGT—gamma glutamyltransferase, α-AA—α-Amino acids, SOD—superoxide dismutase, SM—seromucoids.

When taking into account the expression of progesterone receptors, it was shown that PR-positive and PR-negative subgroups practically did not differ from each other, but significantly differed from the control group (*p < 0.0001*, Figure 7A). Albumin (*r = 0.7587*), total protein (*r = 0.6834*), seromucoids (*r = 0.6816*), catalase (*r = 0.6092*), GGT (*r = 0.5642*), ALP (*r= 0.5552*), LDH (*r = 0.5456*), α-amino acids (*r = 0.5192*), urea (*r = 0.4215*), and α-amylase (*r = 0.4009*) made the main contributions to the separation by PC1. Separation by PC2 was due to the contribution of urea (*r = 0.5290*), SOD/Catalase-ratio (*r = 0.4955*), α-amino acids (*r = 0.4829*), and catalase (*r = -0.4482*) (Figure 7B). When taking into account the degree of expression of progesterone receptors, it was shown that the vertical axis separated the PR (−), PR (+), and PR (++) groups from the PR (+++) group and the control group (*p = 0.0048*, Figure 7C). The horizontal axis separated patients with breast cancer from controls (*p<0.0001*). The contribution of biochemical indicators to the division of subgroups practically did not change (Figure 7B vs. Figure 7D).

**Figure 8:**
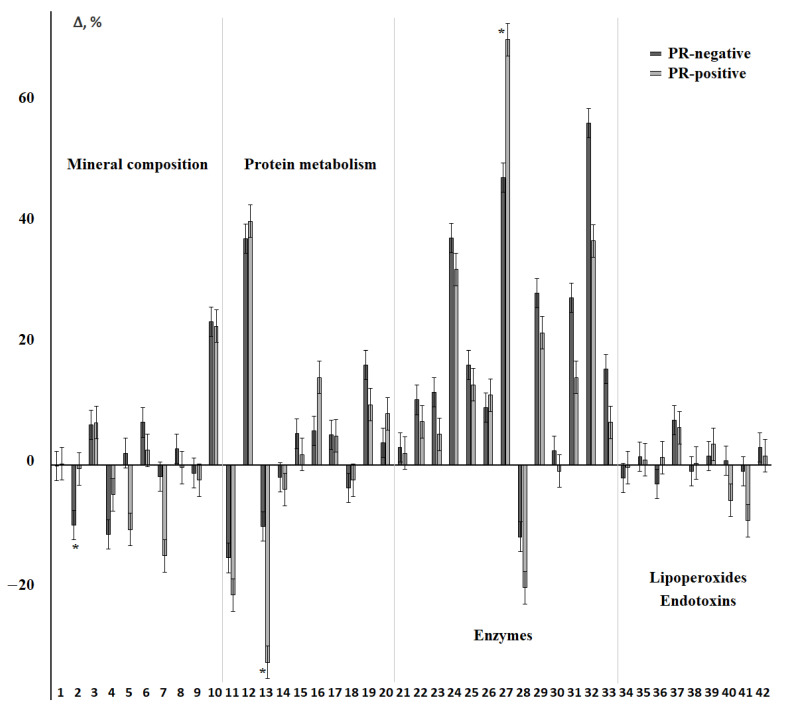
Relative change in biochemical indicators of saliva depending on the status of PR receptors. The interval of variation is given in comparison with the control group. The numbers of biochemical indicators correspond to the serial number in Table 2 and Table 3. *—differences between groups with PR-positive and PR-negative status are statistically significant, *p* < 0.05.

Differences between PR-positive and PR-negative breast cancer were statistically significant for calcium, uric acid, and α-amylase (Figure 8, Supplementary Table S7). The content of sodium, chlorides, AOA, Schiff bases, and MM 254 and 280 nm changed in different directions compared to the control group (Figure 8).

Differences between PR (+) and PR (++) were significant in terms of ALP activity (−17.9%, *p = 0.0353*), catalase (−29.1%, *p = 0.0125*), and seromucoids level (−18.6%, *p = 0.0196*) (Supplementary Table S8). The same parameters determined the difference between the PR (+) and PR (+++) groups; however, the content of protein, albumin, AST/ALT-ratio, GGT, and α-amylase were also added. All of the listed indicators showed a decrease in values for the PR (+) and PR (+++) groups.

Simultaneous consideration of the positive and negative status of estrogen and progesterone receptors gave similar results with division by molecular biological subtypes of breast cancer (Supplementary Table S9). Thus, the ER/PR-negative subgroup united the subgroups of basal-like and non-luminal breast cancer, while the ER/PR-positive subgroup united the luminal subtypes of breast cancer (Supplementary Tables S1 and S2). Differences between ER/PR-negative and ER/PR-positive breast cancer were identified in the content of calcium (−10.0 and 0.0%), sodium (−2.2 and −11.4%), and MDA (+25, 6 and +6.4%), as well as the activity of ALP (+31.0% and +13.8%) and peroxidase (+57.5 and +17.8% for ER/PR-negative and ER/PR-positive breast cancer respectively. Changes are shown compared to the control group).

## 4. Discussion

Most studies on the analysis of saliva in breast cancer were aimed at identifying biomarkers that can differentiate patients with breast cancer from healthy controls [28,29,30,31,32,33,34,35,36,37,38,39,40,41,42,43,44]. Previously, we identified 11 metabolites that allow us to do this with a sensitivity of up to 91% [47]. These indicators included urea, total protein, total content of α-amino acids, MDA, NO, Na/K-ratio, SB/TC-ratio, as well as ALP and GGT activity. When taking into account the molecular biological subtype of breast cancer, Na/K-ratio and SB/TC-ratio do not contribute to the division of subgroups; however, α-amylase, LDH, catalase, SOD/Catalase-ratio, and SOD/Peroxidase-ratio become significant (Table 3). Within the breast cancer group, we showed the maximum differences for basal-like cancer in terms of increased levels of endogenous toxins (MM 254 and 280) and lipid peroxidation products (DC, MDA), as well as SOD/Catalase. Complex metabolic disorders and nonspecific clinical manifestations that accompany the development of malignant neoplasms are characterized as endogenous intoxication syndrome [52,53,54]. An increase in the ratio of MM 280/254 nm is indirect evidence of the excessive generation of active oxygen metabolites, superoxide radicals, and hydrogen peroxide [55]. Hydroxyl radicals are capable of damaging the phosphoglyceride membrane structures of cell membranes and their organoids. The object of exposure to active oxygen metabolites is arachidonic acid containing four double bonds separated by CH_2_ groups. When exposed to hydroxyl radicals, double bonds become conjugated and diene conjugates are formed, which later turn into lipid hydroperoxides [51,56]. This situation reflects the fact that the accumulation of endogenous toxins and lipid peroxides occurs at a faster rate than their inactivation by the antioxidant defense system. It is significant that such a picture was observed for the BC subtype, which has the least favorable prognosis [57]. Differences in the largest number of indicators were found for basal-like and luminal A subtypes of breast cancer. For other subtypes of breast cancer, significant differences were found only in comparison with the control group.

Based on data reported in a study [46], the levels of five metabolites differed significantly between the luminal A-like and B-like subtypes (cadaverine, 5-aminovalerate, gamma-butyrobetaine, 2-hydroxy-4-methylpentanoate, alanine), while N-acetylneuraminate was only significantly differentiating between the luminal A-like and triple negative subtypes. For other metabolites, no differences were found between breast cancer subtypes [46]. The only indirect intersection in the list of determined parameters refers to alanine, since this amino acid is included in the indicator of the total content of α-amino acids determined by us. Nevertheless, both studies confirm that there are metabolic features of saliva depending on the molecular biological subtype of breast cancer, which shows the promise of research in this direction. The need for research is confirmed by the fact that it is not always possible to determine the molecular biological subtype of a tumor. Thus, in our sample, 13.1% of patients with breast cancer lack the results of immunohistochemistry of the tumor, which imposes certain restrictions on the choice of treatment tactics and determining the prognosis of the disease.

We tried to analyze the influence of each factor that determines the assignment to one or another subtype of breast cancer separately. The results obtained have not been described previously in the literature. Thus, it is considered that patients whose samples were assessed as HER2 (+++) have a positive HER2 status, and HER2 (−)/(+) have a negative status. HER2 (++) samples are considered indeterminate and should be retested by in situ hybridization. According to our data, samples with HER2 (−) and HER2 (+) status had no differences (Figure 3A,C) and were singled out on the factor diagram by one field, which once again confirms the legitimacy of considering these subgroups as one HER2-negative groups. Differences with the control group in this case were expressed as much as possible. The most important biochemical indicators that determine the division into HER2-positive and HER2-negative subgroups were the metabolic enzymes ALT and AST, as well as uric acid and the SOD/catalase-ratio.

Changes in the biochemical composition of saliva at different levels of estrogen and progesterone receptors were more pronounced. For ER-positive breast cancer, the salivary composition was significantly different from the control, but the differences between ER-positive and ER-negative BC were noticeable. Significant differences were found in terms of lipid peroxidation (DC, MDA), peroxidase activity, SOD/Peroxidase-ratio, ALP, and LDH. It is known that salivary peroxidase plays a dual role: it is responsible for the breakdown of cytotoxic hydrogen peroxide and has bactericidal activity against the oral microbiota [58]. Salivary peroxidase is the only antioxidant synthesized exclusively in the salivary glands [59]. Thus, salivary peroxidase activity reflects the effectiveness of the salivary glands in preventing oxidative stress. An increase in peroxidase activity in the saliva of patients with breast cancer indicates an increase in the enzymatic antioxidant defense that protects the salivary glands and the entire oral cavity from oxidative damage. An increase in peroxidase activity occurred against the background of an increase in the level of lipid peroxidation products and was characteristic of ER-negative breast cancer, which has the least favorable prognosis. It is known that ER-regulated overexpression of the HER2 protein is combined with increased activity in the tumor of the muscle isoform of LDH, one of the key enzymes of the glycolytic pathway of glucose oxidation, while LDH activity was higher in the blood of patients with ER-negative tumors [60]. Previously, we showed the existence of a correlation between the activity of LDH in saliva and blood plasma [61]. For PR receptors, no differences between PR-positive and PR-negative breast cancer in saliva were found. When considering combinations of the level of ER and PR receptors, it was shown that for ER/PR-positive breast cancer in saliva, the activity of metabolic enzymes (ALP, LDH) was statistically lower, the level of lipid peroxides (DC, MDA) was lower, as was the content of uric acid, catalase, and peroxidases. This indicates a less pronounced intensity of lipid peroxidation processes and a balanced work of the antioxidant defense system (both its enzymatic and non-enzymatic links). Since only about 10% of all breast cancers have the status of ER (+)/PR (−) and about 5% of ER (−)/PR (+), we did not consider such combinations due to the small number of patients in each [62]. There are data in the literature on the direct determination of HER2 in saliva [63,64,65]. Current research suggests that soluble fragments of the HER2 oncogene may be released from the cell surface and found in patients with breast carcinoma. The salivary HER2 protein assay has been shown to be reliable and may have potential applications in the initial detection and subsequent screening of recurrent breast cancer [63]. HER2 has been found in the saliva of women with benign breast lesions and women diagnosed with breast cancer. HER2 levels in cancer patients were significantly higher than those in saliva of healthy controls and patients with benign tumors [64,65]. Nevertheless, there are a number of unanswered questions, including how the level of HER2 in saliva correlates with its content in the tumor tissue and whether the level of HER2 allows the tumor to be assigned to a specific molecular biological subtype, which determines the choice of treatment tactics. It should be noted that the correlation between salivary hormonal status and tumor receptor status has also not been proven; therefore, tumor biopsy is still a necessary step in diagnosis and treatment. However, for treatment progression and recurrence monitoring, the choice of indirect salivary indicators associated with a particular tumor type may be important.

The limitations of the study were related to the fact that we initially chose metabolites that can be determined using a biochemical analyzer. At this stage, we have shown potential directions for research; in particular, the important role of the total content of α-amino acids shows the need to determine the amino acid profile. In continuation of the research, we plan to analyze the ALP and LDH isoenzymes. The limitations included the fact that we did not conduct a parallel determination of HER2 in saliva and did not assess the hormonal status of saliva. In this work, we did not determine the prognostic significance of the selected saliva biochemical indicators and did not evaluate their change during treatment.

## 5. Conclusions

Our study showed that various molecular biological subtypes of breast cancer are characterized by changes in the metabolic profile of saliva. It was shown that the composition of the saliva of patients with basal-like breast cancer differed from the control group as much as possible. The biochemical composition of saliva varies more depending on the HER2 status and the status of estrogen receptors and, to a lesser extent, on the status of progesterone receptors. It was found that the HER2 (−)/HER2 (+) group, which should be considered as a single group, as well as ER-positive breast cancer, differed statistically significantly from the control group. For ER/PR-positive breast cancer, a more favorable ratio of biochemical indicators of saliva was also noted. Thus, the composition of saliva reacts very subtly to changes in the human body, including the ability to assess metabolic changes in different molecular biological subtypes of breast cancer. All this emphasizes the prospects for continuing research in this direction.

## Figures and Tables

**Table 1:** The structure of the study group.

Feature	Breast Cancer, *n* = 487	Control Group, *n* = 298
Age, years	54.5 [47.0; 56.0]	49.3 [43.8; 56.1]
Histological type		
Ductal	227 (46.6%)	-
Lobular	86 (17.7%)	-
Mixed (Ductal + Lobular)	12 (2.5%)	-
Rare forms	58 (11.9%)	-
Unknown	104 (21.3%)	-
Clinical Stage		
Stage I	119 (24.4%)	-
Stage IIa	123 (25.3%)	-
Stage IIb	88 (18.1%)	-
Stage IIIa	55 (11.3%)	-
Stage IIIb	47 (9.6%)	-
Stage IV	55 (11.3%)	-
Subtype		
Luminal A-like	64 (13.1%)	-
Luminal B-like (HER2+)	230 (47.4%)	-
Luminal B-like (HER2−)	63 (12.9%)	-
Non-Luminal (HER2+)	38 (7.8%)	-
Basal-like (Triple-negative)	28 (5.7%)	-
Unknown	64 (13.1%)	-
HER2-status		
HER2-negative HER2 (−)	156 (36.1%)	-
HER2-positive	276 (63.9%)	-
HER2 (+)	124 (44.9%)	-
HER2 (++)	83 (30.1%)	-
HER2 (+++)	69 (25.0%)	-
ER-status		
ER-negative ER (−)	77 (17.7%)	-
ER-positive	359 (82.3%)	-
ER (+)	60 (16.7%)	-
ER (++)	77 (21.4%)	-
ER (+++)	222 (61.9%)	-
PR-status		
PR-negative PR (−)	125 (28.7%)	-
PR-positive	310 (71.3%)	-
PR (+)	64 (20.6%)	-
PR (++)	79 (25.5%)	-
PR (+++)	167 (53.9%)	-

**Table 2:** Changes in biochemical indicators of saliva compared with the control group, %.

No.	Indicators	Lum A	Lum B (+)	Lum B (−)	BL	Non-Lum	Kruskal–Wallis Test (H, *p*)
1	pH	0.6	−0.1	−0.2	1.1	−1.3	4.125; 0.5316
2	Calcium, mmol/L	8.7	−4.2	−1.2	−11.5	−9.7	6.424; 0.2671
3	Phosphorus, mmol/L	0.7	7.5	8.7	9.7	−5.4	7.439; 0.1900
4	Ca/P-ratio, c.u.	0.5	−8.4	−12.4	−20.2	−6.0	8.226; 0.1442
5	Sodium, mmol/L	−21.4	−12.5	7.9	5.0	−15.2	6.519; 0.2589
6	Potassium, mmol/L	−1.3	2.1	21.6	23.0	5.3	7.879; 0.1631
7	Na/K-ratio, c.u.	−15.5	−14.0	−13.4	−17.0	3.3	5.517; 0.3560
8	Chlorides, mmol/L	−3.9	−2.0	6.6	12.8	−2.1	7.662; 0.1759
9	Magnesium, mmol/L	−6.5	−0.7	0.1	−7.4	−1.4	1.992; 0.8503
10	NO, μmol/L	22.4	21.6	40.7	19.0	49.3	16.02; 0.0068 *
11	Protein, mg/mL	−24.5	−21.4	−12.8	−5.7	−10.7	70.13; 0.0000 *
12	Urea, mmol/L	33.0	42.6	46.5	41.0	36.0	66.45; 0.0000 *
13	Uric acid, μmol/L	−28.8	−34.0	−21.9	2.6	−9.2	7.819; 0.1665
14	Lactic acid, mmol/L	10.4	−3.0	−6.4	−1.5	−4.9	4.204; 0.5205
15	Pyruvic acid, μmol/L	−1.8	1.8	10.7	8.9	5.4	4.440; 0.4879
16	Albumin, mg/mL	14.7	8.8	40.1	0.5	29.5	6.786; 0.2371
17	α-Aminoacids, mmol/L	3.8	4.8	9.1	6.5	5.2	29.84; 0.0000 *
18	Imidazole compounds, mmol/L	−7.9	−3.9	13.2	3.9	−2.6	6.158; 0.2912
19	Sialic acids, mmol/L	0.0	13.8	13.8	37.9	−5.2	7.038; 0.2179
20	Seromucoids, c.u.	8.8	6.6	8.2	8.8	2.7	6.123; 0.2944
21	ALT, U/L	2.0	10.0	6.0	10.0	16.0	0.9695; 0.9650
22	AST, U/L	16.4	10.4	16.4	26.9	10.4	9.703; 0.0841
23	AST/ALT-ratio, c.u.	16.7	1.4	19.0	26.0	11.8	8.854; 0.1150
24	LDH, U/L	43.1	31.8	4.6	21.3	65.3	18.78; 0.0021 *
25	ALP, U/L	13.8	6.9	44.8	34.7	24.1	21.68; 0.0006 *
26	GGT, U/L	8.7	12.3	17.3	13.2	2.4	40.03; 0.0000 *
27	Catalase, nkat/mL	−14.9	−22.5	7.7	-18.2	−10.8	15.24; 0.0094 *
28	Superoxide dismutase, c.u.	2.3	31.8	45.5	27.3	15.9	10.79; 0.0557
29	α-Amylase, U/L	60.7	65.1	141.0	60.5	20.6	17.41; 0.0038 *
30	Antioxidant activity, mmol/L	−14.1	−0.8	4.9	2.4	−11.0	2.688; 0.7479
31	Peroxidase, c.u.	−9.6	9.6	16.4	20.5	86.3	7.194; 0.2066
32	SOD/Catalase-ratio, c.u.	3.2	53.6	53.3	80.3	36.9	12.12; 0.0332 *
33	SOD/Peroxidase-ratio, c.u.	−9.6	12.4	88.9	51.8	−35.0	13.97; 0.0158 *
34	Diene conjugates, c.u.	−3.1	−0.3	−2.8	1.7	0.7	8.617; 0.1254
35	Triene conjugates, c.u.	6.6	−0.7	−0.9	1.1	2.0	3.988; 0.5511
36	Schiff bases, c.u.	10.2	0.6	−3.2	−3.3	3.1	7.175; 0.2079
37	MDA, μmol/L	1.3	6.4	9.6	35.9	4.5	22.95; 0.0003 *
38	SB/(DC+TC)-ratio, c.u.	5.9	−0.4	−2.2	−0.7	−0.2	9.043; 0.1074
39	SB/TC-ratio, c.u.	7.2	1.6	−1.1	3.0	7.2	10.05; 0.0739
40	MM 254, c.u.	−18.8	−5.4	5.4	24.1	1.7	7.962; 0.1583
41	MM 280, c.u.	−16.7	−8.0	−10.3	22.0	−5.9	6.170; 0.2901
42	MM 280/254	2.8	0.9	3.1	4.5	1.0	4.729; 0.4498

Note. *—differences with the control group are statistically significant at *p* < 0.05.

**Table 3:** Values of the Kruskal–Wallis test when comparing subgroups by the level of expression of HER2, estrogen receptors and progesterone, taking into account the control group.

No.	Indicators	Kruskal–Wallis Test (H, *p*) + Control Group		
		HER2	ER	PR
1	pH	2.858; 0.5818	3.467; 0.4829	0.7895; 0.9399
2	Calcium, mmol/L	5.365; 0.2518	8.055; 0.0896 **	7.971; 0.0926 **
3	Phosphorus, mmol/L	6.013; 0.1982	9.168; 0.0570 **	3.688; 0.4498
4	Ca/P-ratio, c.u.	6.397; 0.1714	5.822; 0.2128	6.498; 0.1649
5	Sodium, mmol/L	3.038; 0.5515	4.154; 0.3856	3.286; 0.5112
6	Potassium, mmol/L	5.475; 0.2419	1.370; 0.8494	1.119; 0.8913
7	Na/K-ratio, c.u.	5.651; 0.2268	3.911; 0.4182	6.548; 0.1618
8	Chlorides, mmol/L	4.632; 0.3272	5.517; 0.2382	5.454; 0.2483
9	Magnesium, mmol/L	0.6162; 0.9612	1.312; 0.8593	0.6850; 0.9532
10	NO, μmol/L	16.04; 0.0030 *	17.23; 0.0017 *	20.47; 0.0004 *
11	Protein, mg/mL	75.09; 0.0000 *	89.55; 0.0000 *	79.20; 0.0000 *
12	Urea, mmol/L	65.41; 0.0000 *	66.16; 0.0000 *	65.78; 0.0000 *
13	Uric acid, μmol/L	8.990; 0.0614 **	11.65; 0.0202 *	9.063; 0.0596 **
14	Lactic acid, mmol/L	4.819; 0.3064	2.271; 0.6860	4.215; 0.3776
15	Pyruvic acid, μmol/L	5.060; 0.2812	2.375; 0.6672	4.344; 0.3614
16	Albumin, mg/mL	8.648; 0.0705 **	7.980; 0.0923 **	12.68; 0.0130 *
17	α-Aminoacids, mmol/L	24.58; 0.0001 *	25.68; 0.0000 *	26.72; 0.0000 *
18	Imidazole compounds, mmol/L	4.478; 0.3452	4.757; 0.3132	4.123; 0.3896
19	Sialic acids, mmol/L	0.7944; 0.9392	2.169; 0.7047	1.640; 0.8017
20	Seromucoids, c.u.	6.399; 0.1713	7.597; 0.1075	10.70; 0.0302 *
21	ALT, U/L	3.192; 0.5263	0.7748; 0.9418	3.865; 0.4245
22	AST, U/L	9.008; 0.0609 **	3.668; 0.4528	5.101; 0.2771
23	AST/ALT-ratio, c.u.	9.652; 0.0467 *	3.293; 0.5101	9.622; 0.0473 *
24	LDH, U/L	13.83; 0.0078 *	14.50; 0.0059 *	12.35; 0.0149 *
25	ALP, U/L	14.46; 0.0060 *	26.72; 0.0000 *	18.83; 0.0008 *
26	GGT, U/L	39.01; 0.0000 *	36.20; 0.0000 *	42.78; 0.0000 *
27	α-Amylase, U/L	17.76; 0.0014 *	16.05; 0.0030 *	19.30; 0.0007 *
28	Catalase, nkat/mL	14.51; 0.0058 *	16.04; 0.0030 *	19.41; 0.0007 *
29	Superoxide dismutase, c.u.	6.113; 0.1908	5.349; 0.2533	6.311; 0.1771
30	Antioxidant activity, mmol/L	1.095; 0.8950	1.206; 0.8772	2.973; 0.5623
31	Peroxidase, c.u.	3.604; 0.4622	5.628; 0.2287	6.264; 0.1803
32	SOD/Catalase-ratio, c.u.	7.923; 0.0944 **	5.605; 0.2307	10.07; 0.0393 *
33	SOD/Peroxidase-ratio, c.u.	5.470; 0.2424	6.314; 0.1769	11.47; 0.0217 *
34	Diene conjugates, c.u.	7.459; 0.1135	9.019; 0.0606 **	2.224; 0.6946
35	Triene conjugates, c.u.	2.874; 0.5791	5.884; 0.2080	2.649; 0.6181
36	Schiff bases, c.u.	2.215; 0.6962	3.841; 0.4280	4.934; 0.2942
37	MDA, μmol/L	16.40; 0.0025 *	19.98; 0.0005 *	19.17; 0.0007 *
38	SB/(DC+TC)-ratio, c.u.	2.115; 0.7146	8.384; 0.0785 **	4.159; 0.3849
39	SB/TC-ratio, c.u.	2.369; 0.6682	5.409; 0.2478	3.029; 0.5529
40	MM 254, c.u.	5.346; 0.2536	8.585; 0.0724 **	6.688; 0.1533
41	MM 280, c.u.	4.588; 0.3323	6.409; 0.1706	5.865; 0.2095
42	MM 280/254	4.545; 0.3372	5.853; 0.2104	4.260; 0.3720

Note. *—differences are statistically significant at *p* < 0.05; **—differences are statistically significant at *p* < 0.10.

## Data Availability

The data presented in this study are available on request from the corresponding author. The data are not publicly available because they are required for the preparation of a Ph.D. Thesis.

## Supplementary Materials

The following supporting information can be downloaded at: https://www.mdpi.com/article/10.3390/cimb44070211/s1, Table S1: Biochemical composition of saliva for Luminal B-like breast cancer compared with the control group. Table S2: Biochemical composition of saliva for Luminal A-like, Non-Luminal and Basal-like breast cancer compared with the control group. Table S3: Biochemical composition of saliva in HER2-positive and HER2-negative breast cancer. Table S4: Biochemical composition of saliva in HER2-positive (+, ++, +++) breast cancer. Table S5: Biochemical composition of saliva in ER-positive and ER-negative breast cancer. Table S6: Biochemical composition of saliva in ER-positive (+, ++, +++) breast cancer. Table S7: Biochemical composition of saliva in PR-positive and PR-negative breast cancer. Table S8: Biochemical composition of saliva in PR-positive (+, ++, +++) breast cancer. Table S9: Biochemical composition of saliva in ER/PR-positive and ER/PR-negative breast cancer.
