# Supplementary material for: «Salivaomics» of Different Molecular Biological Subtypes of Breast Cancer
Source: Curr Issues Mol Biol. 2022 Jul 5;44(7):3053–74. doi: 10.3390/cimb44070211 (PMC9319144; doi:10.3390/cimb44070211)
Supplement: Supplementary file 1 [file cimb-44-00211-s001.zip › cimb-1789930-supplementary.pdf]

Table S1. Biochemical composition of saliva for Luminal B-like breast cancer compared with the control group

Indicator	Control Group, n=298	Luminal B-like (HER2+), n=230	Luminal B-like (HER2-), n=63
pH	6.48 [6.30; 6.71]	6.48 [6.23; 6.70]	6.47 [6.21; 6.73]
Calcium, mmol/L	1.28 [1.04; 1.59]	1.22 [0.86; 1.60]	1.26 [0.86; 1.72]
Phosphorus, mmol/L	4.33 [3.35; 5.72]	4.65 [3.62; 5.94]**	4.71 [4.01; 6.07]*
Ca/P-ratio, c.u.	0.287 [0.220; 0.389]	0.263 [0.191; 0.341]*	0.252 [0.182; 0.387]
Sodium, mmol/L	8.35 [5.55; 12.09]	7.31 [4.57; 12.14]	9.01 [5.87; 17.03]
Potassium, mmol/L	10.76 [8.76; 14.20]	10.99 [8.18; 14.06]	13.08 [10.37; 14.79]**
Na/K-ratio, c.u.	0.751 [0.505; 1.190]	0.646 [0.466; 0.970]**	0.650 [0.467; 1.140]
Chlorides, mmol/L	25.71 [20.93; 31.50]	25.20 [20.22; 31.49]	27.42 [21.65; 36.40]
Magnesium, mmol/L	0.302 [0.242; 0.360]	0.300 [0.227; 0.371]	0.302 [0.237; 0.432]
NO, µmol/L	23.51 [13.51; 40.70]	28.60 [16.84; 45.96]*	33.07 [17.37; 50.00]*
Protein, mg/mL	0.79 [0.49; 1.23]	0.62 [0.38; 1.02]*	0.68 [0.37; 1.02]
Urea, mmol/L	6.86 [4.52; 9.47]	9.78 [6.03; 13.35]*	10.04 [7.23; 16.28]*
Uric acid, µmol/L	90.38 [34.62; 151.38]	59.62 [23.08; 133.03]*	70.56 [21.93; 145.24]
Lactic acid, mmol/L	1.70 [1.31; 2.03]	1.65 [1.40; 2.01]	1.60 [1.38; 1.96]
Pyruvic acid, µmol/L	13.73 [9.56; 19.85]	13.97 [9.80; 19.00]	15.20 [10.29; 21.32]
Albumin, mg/mL	0.270 [0.182; 0.459]	0.294 [0.181; 0.510]	0.378 [0.197; 0.653]*
α-Aminoacids, mmol/L	4.03 [3.75; 4.31]	4.22 [3.92; 4.81]*	4.40 [3.95; 5.09]*
Imidazole compounds, mmol/L	0.288 [0.182; 0.395]	0.277 [0.182; 0.395]	0.326 [0.175; 0.478]
Sialic acids, mmol/L	0.177 [0.128; 0.275]	0.201 [0.146; 0.281]**	0.201 [0.153; 0.275]
Seromucoids, c.u.	0.091 [0.062; 0.134]	0.097 [0.059; 0.145]	0.099 [0.069; 0.179]*
ALT, U/L	3.85 [2.77; 5.08]	4.23 [3.00; 5.62]*	4.08 [3.08; 4.92]
AST, U/L	5.58 [3.58; 7.50]	6.17 [4.33; 8.67]*	6.50 [4.58; 8.08]*
AST/ALT-ratio, c.u.	1.38 [1.11; 1.85]	1.40 [1.14; 1.83]	1.65 [1.22; 2.09]*
LDH, U/L	1076.0 [599.5; 1875.0]	1418.0 [837.7; 2022.0]*	1126.0 [642.3; 2117.0]
ALP, U/L	63.02 [43.46; 91.27]	67.36 [45.63; 97.79]	91.27 [54.33; 123.86]*
GGT, U/L	20.8 [17.6; 24.4]	23.4 [20.3; 26.1]*	24.4 [19.8; 27.0]*
Catalase, nkat/mL	4.44 [3.38; 5.69]	3.44 [2.46; 5.66]*	4.78 [2.46; 6.85]
Superoxide dismutase, c.u.	57.89 [31.58; 105.26]	76.32 [34.21; 157.89]*	84.21 [38.16; 130.26]*
α-Amylase, U/L	185.2 [83.5; 384.4]	305.7 [122.6; 564.8]*	446.2 [244.7; 833.0]*
Antioxidant activity, mmol/L	2.36 [1.53; 3.65]	2.34 [1.49; 3.53]	2.48 [1.71; 3.38]
Peroxidase, c.u.	0.365 [0.170; 0.670]	0.400 [0.230; 0.830]	0.425 [0.230; 0.900]
SOD/Catalase-ratio, c.u.	14.29 [6.62; 28.70]	21.95 [9.08; 43.46]*	21.91 [9.84; 38.73]*
SOD/Peroxidase-ratio, c.u.	122.60 [60.31; 246.71]	137.84 [65.19; 296.05]	231.64 [101.21; 442.11]*
Diene conjugates, c.u.	3.95 [3.80; 4.11]	3.94 [3.71; 4.13]	3.84 [3.73; 4.07]*
Triene conjugates, c.u.	0.886 [0.793; 0.971]	0.879 [0.797; 1.000]	0.878 [0.805; 0.985]
Schiff bases, c.u.	0.537 [0.493; 0.570]	0.540 [0.483; 0.623]	0.519 [0.476; 0.609]
MDA, µmol/L	6.67 [5.73; 8.21]	7.09 [5.81; 8.63]**	7.31 [6.07; 9.83]*
SB/(DC+TC)-ratio, c.u.	0.111 [0.104; 0.116]	0.111 [0.102; 0.124]	0.109 [0.100; 0.124]
SB/TC-ratio, c.u.	0.603 [0.560; 0.656]	0.613 [0.559; 0.679]	0.597 [0.537; 0.686]
MM 254, c.u.	0.261 [0.175; 0.381]	0.247 [0.167; 0.364]	0.275 [0.166; 0.410]
MM 280, c.u.	0.219 [0.147; 0.321]	0.201 [0.142; 0.319]	0.196 [0.135; 0.337]
MM 280/254	0.843 [0.757; 0.949]	0.850 [0.775; 0.951]	0.869 [0.783; 0.953]

Note. * - differences with the control group are statistically significant at p<0.05; ** - differences with the control group are statistically significant at p<0.10.

Table S2. Biochemical composition of saliva for Luminal A-like, Non-Luminal and Basal-like breast cancer compared with the control group

Indicator	Luminal A-like, n=64	Non-Luminal (HER2+), n=38	Basal-like (Triple-negative), n=28
pH	6.52 [6.26; 6.84]	6.40 [6.20; 6.68]	6.55 [6.43; 6.75]
Calcium, mmol/L	1.39 [0.95; 1.65]	1.15 [0.92; 1.40]*	1.13 [0.73; 1.82]
Phosphorus, mmol/L	4.36 [3.22; 6.04]	4.10 [2.49; 5.51]	4.75 [3.09; 7.49]
Ca/P-ratio, c.u.	0.289 [0.199; 0.405]	0.270 [0.210; 0.371]	0.229 [0.156; 0.342]*
Sodium, mmol/L	6.56 [4.27; 12.39]**	7.08 [5.31; 12.51]	8.77 [6.46; 13.02]
Potassium, mmol/L	10.63 [7.43; 15.43]	11.34 [4.50; 13.54]	13.24 [10.18; 17.66]*
Na/K-ratio, c.u.	0.635 [0.492; 0.975]	0.776 [0.489; 1.255]	0.624 [0.408; 1.085]
Chlorides, mmol/L	24.72 [18.08; 32.24]	25.17 [21.10; 30.80]	29.01 [24.76; 35.56]*
Magnesium, mmol/L	0.282 [0.210; 0.372]	0.298 [0.209; 0.398]	0.280 [0.194; 0.403]
NO, µmol/L	28.77 [21.32; 42.19]*	35.09 [18.60; 44.56]	27.98 [22.63; 38.86]
Protein, mg/mL	0.59 [0.32; 1.36]	0.70 [0.36; 1.08]	0.74 [0.37; 1.18]
Urea, mmol/L	9.12 [5.95; 14.02]*	9.32 [5.25; 11.28]*	9.67 [6.61; 13.56]*
Uric acid, µmol/L	64.36 [32.22; 130.56]	82.05 [30.37; 133.33]	92.71 [17.00; 155.65]
Lactic acid, mmol/L	1.88 [1.43; 2.22]	1.62 [1.39; 2.14]	1.68 [1.38; 2.20]
Pyruvic acid, µmol/L	13.48 [8.58; 17.89]	14.46 [10.78; 20.59]	14.95 [12.50; 22.06]
Albumin, mg/mL	0.310 [0.149; 0.494]	0.350 [0.154; 0.590]	0.271 [0.197; 0.530]
α-Aminoacids, mmol/L	4.18 [3.83; 4.74]*	4.24 [3.84; 4.76]**	4.30 [3.86; 5.28]*
Imidazole compounds, mmol/L	0.266 [0.167; 0.357]	0.281 [0.235; 0.349]	0.300 [0.224; 0.391]
Sialic acids, mmol/L	0.177 [0.134; 0.305]	0.168 [0.098; 0.244]	0.244 [0.171; 0.299]**
Seromucoids, c.u.	0.099 [0.058; 0.171]	0.094 [0.064; 0.156]	0.099 [0.076; 0.168]
ALT, U/L	3.92 [2.46; 5.77]	4.46 [2.62; 5.77]	4.23 [3.31; 8.92]*
AST, U/L	6.50 [4.50; 9.67]*	6.17 [4.17; 8.50]	7.08 [5.83; 9.92]*
AST/ALT-ratio, c.u.	1.61 [1.22; 1.96]**	1.55 [1.07; 2.17]	1.74 [1.15; 2.07]
LDH, U/L	1540.0 [1118.0; 2114.5]*	1778.5 [1128.0; 2236.0]*	1305.0 [832.8; 2121.0]
ALP, U/L	71.71 [47.81; 102.13]	78.23 [54.33; 108.65]*	84.86 [49.98; 148.85]*
GGT, U/L	22.6 [19.7; 26.2]*	21.3 [19.1; 24.6]	23.6 [20.6; 27.6]*
Catalase, nkat/mL	3.78 [2.68; 5.22]*	3.96 [2.89; 6.39]	3.63 [2.10; 6.03]**
Superoxide dismutase, c.u.	59.21 [21.05; 86.84]	67.11 [42.11; 115.79]	73.68 [50.00; 165.79]**
α-Amylase, U/L	297.6 [116.5; 579.0]*	223.2 [117.6; 649.8]	297.2 [115.4; 524.8]
Antioxidant activity, mmol/L	2.03 [1.18; 2.93]**	2.10 [1.54; 3.29]	2.42 [1.55; 3.44]
Peroxidase, c.u.	0.330 [0.210; 0.755]	0.680 [0.470; 1.170]*	0.440 [0.335; 0.780]
SOD/Catalase-ratio, c.u.	14.74 [7.41; 23.95]	19.57 [6.88; 36.96]	25.77 [11.17; 43.90]*
SOD/Peroxidase-ratio, c.u.	110.80 [29.51; 224.17]	79.74 [30.96; 112.34]*	186.07 [69.72; 567.43]
Diene conjugates, c.u.	3.83 [3.65; 4.09]*	3.98 [3.79; 4.25]	4.02 [3.77; 4.24]
Triene conjugates, c.u.	0.944 [0.811; 1.065]*	0.903 [0.810; 0.990]	0.895 [0.769; 1.011]
Schiff bases, c.u.	0.591 [0.475; 0.762]*	0.553 [0.492; 0.735]**	0.519 [0.475; 0.683]
MDA, µmol/L	6.75 [5.64; 7.86]	6.97 [6.03; 10.00]	9.06 [6.75; 9.91]*
SB/(DC+TC)-ratio, c.u.	0.118 [0.104; 0.160]*	0.111 [0.102; 0.139]	0.110 [0.099; 0.124]
SB/TC-ratio, c.u.	0.647 [0.573; 0.731]*	0.647 [0.579; 0.727]*	0.622 [0.560; 0.736]
MM 254, c.u.	0.212 [0.139; 0.338]*	0.266 [0.167; 0.426]	0.324 [0.210; 0.443]
MM 280, c.u.	0.182 [0.129; 0.322]**	0.206 [0.157; 0.352]	0.267 [0.177; 0.398]
MM 280/254	0.867 [0.789; 1.017]	0.851 [0.768; 0.940]	0.880 [0.780; 0.963]

Note. * - differences with the control group are statistically significant at $p<0.05$; ** - differences with the control group are statistically significant at $p<0.10$.

Table S3. Biochemical composition of saliva in HER2-positive and HER2-negative breast cancer

Indicator	HER2-negative, n=156	p-value HER2-neg vs. Control	HER2-positive, n=276	p-value HER2-pos vs. Control	p-value HER2-neg vs. HER2-pos
pH	6.50 [6.22; 6.76]	0.4551	6.45 [6.22; 6.69]	0.2622	0.1374
Calcium, mmol/L	1.28 [0.92; 1.75]	0.8454	1.20 [0.86; 1.56]	0.0236*	0.0694**
Phosphorus, mmol/L	4.68 [3.47; 6.13]	0.0767**	4.53 [3.54; 5.87]	0.3180	0.3295
Ca/P-ratio, c.u.	0.27 [0.19; 0.39]	0.1171	0.26 [0.19; 0.35]	0.0051*	0.5106
Sodium, mmol/L	8.1 [4.9; 13.1]	0.4576	7.4 [4.7; 12.5]	0.1468	0.6763
Potassium, mmol/L	12.1 [9.1; 15.2]	0.1710	11.0 [8.1; 14.1]	0.5298	0.0956**
Na/K-ratio, c.u.	0.63 [0.47; 1.04]	0.0825**	0.69 [0.47; 1.08]	0.2059	0.6118
Chlorides, mmol/L	27.4 [19.5; 35.2]	0.2235	25.1 [20.2; 31.5]	0.6884	0.1702
Magnesium, mmol/L	0.293 [0.217; 0.390]	0.8280	0.298 [0.227; 0.371]	0.7018	0.8929
NO, µmol/L	30.0 [21.1; 46.3]	0.0070*	28.6 [16.8; 46.0]	0.0215*	0.5521
Protein, mg/mL	0.64 [0.35; 1.10]	0.0320*	0.61 [0.37; 1.02]	0.0026*	0.9007
Urea, mmol/L	9.60 [6.74; 14.29]	0.0000*	9.78 [6.02; 13.07]	0.0000*	0.2925
Uric acid, µmol/L	67.61 [25.25; 142.50]	0.1053	59.62 [22.94; 132.50]	0.0006*	0.2399
Lactic acid, mmol/L	1.68 [1.38; 2.14]	0.5562	1.63 [1.41; 2.03]	0.9119	0.4967
Pyruvic acid, µmol/L	14.58 [10.05; 19.61]	0.4608	13.97 [9.56; 19.36]	0.9929	0.4726
Albumin, mg/mL	0.312 [0.182; 0.590]	0.1646	0.296 [0.178; 0.514]	0.5986	0.3815
α-Aminoacids, mmol/L	4.30 [3.89; 4.94]	0.0000*	4.22 [3.90; 4.78]	0.0000*	0.8634
Imidazole compounds, mmol/L	0.281 [0.175; 0.417]	0.9141	0.281 [0.190; 0.395]	0.6311	0.7906
Sialic acids, mmol/L	0.201 [0.140; 0.281]	0.1173	0.198 [0.140; 0.272]	0.1867	0.6845
Seromucoids, c.u.	0.100 [0.069; 0.176]	0.0239*	0.096 [0.061; 0.142]	0.5072	0.1104
ALT, U/L	4.00 [3.08; 5.46]	0.0955**	4.08 [2.92; 5.50]	0.0182*	0.8454
AST, U/L	6.71 [4.58; 8.83]	0.0002*	6.17 [4.25; 8.58]	0.0037*	0.2239
AST/ALT-ratio, c.u.	1.65 [1.22; 2.04]	0.0052*	1.43 [1.13; 1.89]	0.6235	0.0200*
LDH, U/L	1449.0 [832.8; 2114.5]	0.0035*	1454.0 [885.3; 2039.0]	0.0005*	0.9183
ALP, U/L	80.40 [47.81; 117.34]	0.0022*	69.54 [45.63; 99.96]	0.2070	0.0487*
GGT, U/L	23.4 [19.9; 27.2]	0.0000*	23.2 [20.2; 26.0]	0.0000*	0.5051
Catalase, nkat/mL	4.06 [2.48; 6.13]	0.0822**	3.46 [2.53; 5.71]	0.0000*	0.1752
Superoxide dismutase, c.u.	73.7 [36.8; 123.7]	0.0677**	73.7 [34.2; 147.4]	0.0077*	0.5667
α-Amylase, U/L	341.4 [143.3; 647.6]	0.0014*	295.8 [120.1; 582.4]	0.0038*	0.4163
Antioxidant activity, mmol/L	2.41 [1.55; 3.34]	0.4052	2.34 [1.49; 3.47]	0.4335	0.7950
Peroxidase, c.u.	0.425 [0.230; 0.800]	0.3032	0.480 [0.280; 0.840]	0.0534**	0.5633
SOD/Catalase-ratio, c.u.	19.4 [8.4; 31.8]	0.0294*	21.1 [8.9; 41.7]	0.0002*	0.2921
SOD/Peroxidase-ratio, c.u.	172.1 [88.3; 404.9]	0.0737**	130.4 [52.2; 265.8]	0.8490	0.0856**
Diene conjugates, c.u.	3.87 [3.71; 4.10]	0.0276*	3.94 [3.72; 4.13]	0.6516	0.1857
Triene conjugates, c.u.	0.909 [0.803; 1.030]	0.0459*	0.884 [0.796; 1.000]	0.4801	0.1794
Schiff bases, c.u.	0.536 [0.476; 0.718]	0.1199	0.540 [0.486; 0.628]	0.0902**	0.5608
MDA, µmol/L	7.09 [5.90; 9.40]	0.0128*	7.09 [5.81; 8.80]	0.0207*	0.5357
SB/(DC+TC)-ratio, c.u.	0.111 [0.100; 0.143]	0.1566	0.111 [0.102; 0.125]	0.2928	0.4592
SB/TC-ratio, c.u.	0.627 [0.553; 0.723]	0.0704**	0.617 [0.561; 0.682]	0.0911**	0.5733
MM 254, c.u.	0.254 [0.164; 0.375]	0.6257	0.245 [0.167; 0.389]	0.3914	0.8722

MM 280, c.u.	0.206 [0.135; 0.337]	0.6851	0.199 [0.141; 0.323]	0.4737	0.9205
MM 280/254	0.867 [0.783; 0.973]	0.0659**	0.850 [0.775; 0.951]	0.3896	0.2090

Note. * - differences are statistically significant at p<0.05; ** - differences are statistically significant at p<0.10.

Table S4. Biochemical composition of saliva in HER2-positive (+, ++, +++) breast cancer

**Indicator**	**HER2(+), n=124**	**HER2(++), n=83**	**HER2(+++), n=69**	**Kruskal-Wallis test (H, p)**
pH	6.45 [6.26; 6.69]	6.45 [6.22; 6.69]	6.43 [6.19; 6.70]	1.634; 0.6517
Calcium, mmol/L	1.20 [0.80; 1.51]	1.23 [1.00; 1.61]	1.14 [0.84; 1.57]	3.957; 0.2662
Phosphorus, mmol/L	4.96 [3.49; 6.00]	4.55 [3.75; 5.55]	4.07 [2.98; 5.48]	4.229; 0.2377
Ca/P-ratio, c.u.	0.25 [0.18; 0.34]	0.27 [0.21; 0.34]	0.27 [0.21; 0.37]	6.507; 0.0894**
Sodium, mmol/L	7.4 [5.1; 11.8]	6.7 [4.5; 12.5]	7.7 [5.4; 12.9]	2.959; 0.3981
Potassium, mmol/L	11.3 [8.8; 14.6]	10.2 [7.4; 13.5]	10.8 [6.9; 13.3]	3.003; 0.3911
Na/K-ratio, c.u.	0.65 [0.47; 0.92]	0.70 [0.47; 1.41]	0.71 [0.47; 1.52]	3.702; 0.2955
Chlorides, mmol/L	24.5 [20.0; 32.5]	24.9 [19.6; 30.4]	26.2 [21.3; 32.3]	2.197; 0.5325
Magnesium, mmol/L	0.292 [0.220; 0.371]	0.304 [0.236; 0.371]	0.298 [0.215; 0.369]	0.5998; 0.8965
NO, µmol/L	33.3 [17.4; 46.0]	26.8 [16.7; 48.1]	27.9 [13.2; 40.4]	10.74; 0.0132*
Protein, mg/mL	0.66 [0.42; 1.12]	0.57 [0.35; 1.02]	0.60 [0.36; 0.98]	65.93; 0.0000*
Urea, mmol/L	9.60 [6.15; 12.96]	9.56 [6.00; 14.18]	9.89 [6.03; 12.27]	47.17; 0.0000*
Uric acid, µmol/L	54.46 [23.44; 130.67]	54.82 [26.21; 126.15]	77.88 [17.43; 134.65]	9.033; 0.0289*
Lactic acid, mmol/L	1.71 [1.41; 2.18]	1.60 [1.31; 1.85]	1.64 [1.47; 1.94]	4.807; 0.1865
Pyruvic acid, µmol/L	14.83 [10.17; 19.73]	12.99 [9.80; 19.12]	12.99 [7.72; 19.00]	3.359; 0.3395
Albumin, mg/mL	0.321 [0.198; 0.607]***	0.249 [0.158; 0.419]	0.298 [0.153; 0.456]	6.445; 0.0919**
α-Aminoacids, mmol/L	4.19 [3.92; 5.06]	4.15 [3.90; 4.63]	4.29 [3.86; 4.81]	19.33; 0.0002*
Imidazole compounds, mmol/L	0.277 [0.193; 0.395]	0.273 [0.152; 0.387]	0.296 [0.193; 0.410]	4.237; 0.2370
Sialic acids, mmol/L	0.192 [0.140; 0.269]	0.191 [0.134; 0.302]	0.204 [0.134; 0.262]	0.2740; 0.9648
Seromucoids, c.u.	0.100 [0.061; 0.160]	0.094 [0.056; 0.133]	0.092 [0.062; 0.133]	1.624; 0.6539
ALT, U/L	4.46 [3.54; 5.77]	3.92 [2.69; 5.30]	3.73 [2.62; 5.00]	3.082; 0.3791
AST, U/L	7.17 [4.67; 9.75]	5.67 [4.17; 7.50]	5.79 [3.96; 8.50]	2.709; 0.4386
AST/ALT-ratio, c.u.	1.44 [1.10; 1.90]	1.35 [1.13; 1.77]	1.48 [1.15; 1.97]	1.449; 0.6940
LDH, U/L	1595.5 [987.1; 2052.5]	1529.0 [781.7; 2019.0]	1347.0 [753.5; 2018.0]	11.53; 0.0092*
ALP, U/L	71.71 [48.89; 102.13]	69.54 [41.29; 99.96]	67.36 [45.63; 97.79]	4.751; 0.1909
GGT, U/L	23.4 [21.1; 26.7]	22.8 [19.8; 24.9]	23.0 [19.8; 26.2]	30.44; 0.0000*
Catalase, nkat/mL	3.80 [2.66; 5.75]	3.33 [2.34; 5.39]	3.24 [2.44; 5.65]	15.44; 0.0015*
Superoxide dismutase, c.u.	71.05 [34.21; 139.47]	65.79 [31.58; 139.47]	81.58 [42.11; 152.63]	5.720; 0.1260
α-Amylase, U/L	423.1 [133.0; 605.3]	242.1 [114.0; 404.2]	297.9 [115.6; 674.7]	11.04; 0.0115*
Antioxidant activity, mmol/L	2.62 [1.61; 3.42]	2.43 [1.18; 3.58]	2.14 [1.59; 3.51]	0.9100; 0.8230
Peroxidase, c.u.	0.560 [0.300; 1.050]	0.370 [0.240; 0.690]	0.480 [0.290; 0.720]	3.755; 0.2892
SOD/Catalase-ratio, c.u.	20.9 [7.0; 36.1]	19.2 [7.4; 41.5]	23.4 [9.9; 51.5]	7.369; 0.0541**
SOD/Peroxidase-ratio, c.u.	129.2 [52.2; 276.7]	160.8 [51.0; 270.3]	106.0 [50.2; 245.6]	1.582; 0.6634
Diene conjugates, c.u.	3.97 [3.79; 4.20]	3.92 [3.70; 4.16]	3.94 [3.68; 4.07]	5.398; 0.1449
Triene conjugates, c.u.	0.897 [0.805; 1.021]	0.865 [0.786; 1.000]	0.858 [0.795; 0.969]	1.566; 0.6672
Schiff bases, c.u.	0.555 [0.496; 0.621]	0.531 [0.476; 0.650]	0.518 [0.483; 0.613]	2.385; 0.4965
MDA, µmol/L	7.35 [5.98; 8.85]	6.92 [5.73; 8.38]	7.09 [5.73; 9.87]	11.84; 0.0079*
SB/(DC+TC)-ratio, c.u.	0.112 [0.101; 0.124]	0.110 [0.102; 0.129]	0.110 [0.102; 0.120]	2.237; 0.5248
SB/TC-ratio, c.u.	0.628 [0.562; 0.682]	0.615 [0.561; 0.684]	0.609 [0.556; 0.679]	1.350; 0.7173
MM 254, c.u.	0.268 [0.170; 0.389]	0.240 [0.150; 0.310]	0.241 [0.167; 0.428]	5.311; 0.1504

MM 280, c.u.	0.217 [0.142; 0.352]	0.189 [0.125; 0.269]	0.180 [0.149; 0.360]	4.608; 0.2029
MM 280/254	0.858 [0.774; 0.958]	0.844 [0.794; 0.937]	0.859 [0.777; 0.954]	1.325; 0.7233

Note. Kruskal-Wallis test values are given to compare four groups: control group, HER2 (+), HER2 (++) and HER2 (+++).
* - differences are statistically significant at p<0.05; ** - differences are statistically significant at p<0.10; *** - differences between HER2 (+) and HER2 (++) are statistically significant, p<0.05.

Table S5. Biochemical composition of saliva in ER-positive and ER-negative breast cancer

Indicator	ER-negative, n=77	p-value ER-neg vs. Control	ER-positive, n=359	p-value ER-pos vs. Control	p-value ER-neg vs. ER-pos
pH	6.46 [6.22; 6.68]	0.4485	6.48 [6.22; 6.73]	0.8982	0.4863
Calcium, mmol/L	1.14 [0.69; 1.44]	0.0103*	1.25 [0.90; 1.64]	0.3553	0.0625*
Phosphorus, mmol/L	4.33 [2.98; 5.95]	0.7185	4.66 [3.57; 5.96]	0.0556**	0.1845
Ca/P-ratio, c.u.	0.26 [0.19; 0.36]	0.0545**	0.27 [0.19; 0.36]	0.0106*	0.6363
Sodium, mmol/L	8.3 [5.5; 12.9]	0.9632	7.6 [4.6; 12.5]	0.1082	0.3395
Potassium, mmol/L	11.6 [9.0; 14.8]	0.6514	11.0 [8.1; 14.2]	0.9953	0.8237
Na/K-ratio, c.u.	0.71 [0.47; 1.26]	0.7949	0.65 [0.47; 1.04]	0.0650**	0.4526
Chlorides, mmol/L	26.3 [21.6; 32.3]	0.5685	25.6 [19.8; 32.2]	0.9170	0.6564
Magnesium, mmol/L	0.282 [0.209; 0.366]	0.3734	0.297 [0.229; 0.377]	0.9227	0.4610
NO, µmol/L	28.0 [16.8; 42.2]	0.0978**	29.1 [17.7; 47.2]	0.0036*	0.8633
Protein, mg/mL	0.64 [0.36; 1.08]	0.0914**	0.62 [0.36; 1.04]	0.0014*	0.8021
Urea, mmol/L	9.66 [6.33; 12.71]	0.0000*	9.61 [6.22; 13.77]	0.0000*	0.6671
Uric acid, µmol/L	80.77 [20.64; 133.33]	0.1130	63.91 [24.75; 136.11]	0.0019*	0.8248
Lactic acid, mmol/L	1.59 [1.39; 2.14]	0.9917	1.66 [1.41; 2.03]	0.8506	0.8928
Pyruvic acid, µmol/L	14.46 [8.82; 20.10]	0.6887	13.97 [9.80; 19.12]	0.8395	0.7519
Albumin, mg/mL	0.286 [0.154; 0.528]	0.8042	0.305 [0.181; 0.560]	0.2616	0.6645
α-Aminoacids, mmol/L	4.24 [3.85; 4.69]	0.0039*	4.23 [3.90; 4.81]	0.0000*	0.3322
Imidazole compounds, mmol/L	0.288 [0.228; 0.379]	0.9206	0.281 [0.175; 0.410]	0.6032	0.6317
Sialic acids, mmol/L	0.204 [0.122; 0.269]	0.6400	0.201 [0.140; 0.281]	0.0653**	0.6340
Seromucoids, c.u.	0.096 [0.073; 0.144]	0.3145	0.098 [0.061; 0.159]	0.1379	0.8826
ALT, U/L	3.92 [2.62; 5.00]	0.9504	3.92 [2.77; 5.15]	0.4691	0.6343
AST, U/L	6.25 [4.00; 8.50]	0.1093	6.00 [4.08; 8.17]	0.0358*	0.7458
AST/ALT-ratio, c.u.	1.69 [1.06; 2.17]	0.0697**	1.47 [1.17; 1.90]	0.1478	0.2023
LDH, U/L	1641.0 [1024.0; 2142.0]	0.0009*	1418.5 [837.2; 2044.0]	0.0013*	0.0788**
ALP, U/L	78.23 [54.33; 119.52]	0.0020*	71.71 [45.63; 104.30]	0.0761**	0.0502**
GGT, U/L	22.8 [20.2; 25.9]	0.0046*	23.3 [20.0; 26.4]	0.0000*	0.3327
Catalase, nkat/mL	3.74 [2.48; 0.13]	0.0228*	3.70 [2.53; 5.80]	0.0001*	0.9633
Superoxide dismutase, c.u.	69.7 [44.7; 117.1]	0.0658**	73.7 [31.6; 142.1]	0.0113*	0.9779
α-Amylase, U/L	228.7 [117.6; 596.8]	0.0937**	318.7 [119.6; 603.7]	0.0003*	0.7395
Antioxidant activity, mmol/L	2.36 [1.49; 3.34]	0.4474	2.34 [1.47; 3.51]	0.3401	0.7781
Peroxidase, c.u.	0.515 [0.330; 0.935]	0.0114*	0.410 [0.230; 0.830]	0.1845	0.0956**
SOD/Catalase-ratio, c.u.	20.7 [7.7; 37.5]	0.0220*	20.8 [8.4; 41.5]	0.0005*	0.8923
SOD/Peroxidase-ratio, c.u.	112.2 [28.6; 186.1]	0.2820	143.9 [68.1; 315.8]	0.1892	0.0445*
Diene conjugates, c.u.	3.99 [3.75; 4.25]	0.2807	3.91 [3.70; 4.11]	0.0501**	0.0358*
Triene conjugates, c.u.	0.905 [0.784; 0.995]	0.5210	0.891 [0.800; 1.016]	0.1766	0.7168

Schiff bases, c.u.	0.530 [0.488; 0.665]	0.2013	0.540 [0.479; 0.652]	0.0804*	0.6228
MDA, µmol/L	8.33 [6.20; 9.96]	0.0003*	7.01 [5.81; 8.72]	0.0407*	0.0194*
SB/(DC+TC)-ratio, c.u.	0.110 [0.100; 0.130]	0.5976	0.111 [0.101; 0.129]	0.1646	0.7925
SB/TC-ratio, c.u.	0.632 [0.574; 0.717]	0.0109*	0.615 [0.556; 0.685]	0.1208	0.1206
MM 254, c.u.	0.275 [0.167; 0.422]	0.6308	0.243 [0.165; 0.369]	0.2330	0.3487
MM 280, c.u.	0.218 [0.146; 0.352]	0.8419	0.197 [0.140; 0.322]	0.3656	0.5111
MM 280/254	0.857 [0.779; 0.948]	0.5785	0.861 [0.783; 0.965]	0.0979**	0.6442

Note. * - differences are statistically significant at $p<0.05$; ** - differences are statistically significant at $p<0.10$.

Table S6. Biochemical composition of saliva in ER-positive (+, ++, +++) breast cancer

**Indicators**	**ER (+), n=60**	**ER (++), n=77**	**ER (+++), n=222**	**Kruskal-Wallis test (H, p)**
pH	6.49 [6.20; 6.75]	6.55 [6.29; 6.79]	6.48 [6.22; 6.70]	2.874; 0.4115
Calcium, mmol/L	1.20 [0.92; 1.67]	1.37 [0.95; 1.77]***	1.24 [0.84; 1.59]	3.208; 0.3606
Phosphorus, mmol/L	4.90 [4.06; 5.94]***	4.68 [3.96; 6.68]***	4.43 [3.45; 5.88]	7.605; 0.0549**
Ca/P-ratio, c.u.	0.24 [0.19; 0.34]	0.28 [0.20; 0.37]	0.27 [0.19; 0.37]	2.703; 0.4396
Sodium, mmol/L	6.9 [5.1; 11.5]	8.1 [5.6; 13.4]	7.4 [4.4; 12.5]	7.157; 0.0671**
Potassium, mmol/L	12.0 [8.5; 14.8]	10.6 [8.2; 14.7]	11.0 [8.0; 14.1]	1.279; 0.7341
Na/K-ratio, c.u.	0.65 [0.43; 0.90]	0.65 [0.52; 1.09]	0.65 [0.47; 1.03]	8.146; 0.0431*
Chlorides, mmol/L	25.3 [20.2; 31.0]	27.8 [21.6; 36.2]***	24.9 [19.2; 31.6]	5.084; 0.1658
Magnesium, mmol/L	0.304 [0.242; 0.367]	0.301 [0.221; 0.372]	0.293 [0.220; 0.383]	0.5012; 0.9186
NO, µmol/L	28.6 [14.0; 48.6]	26.1 [16.6; 48.3]	31.2 [20.5; 43.5]	38.32; 0.0000*
Protein, mg/mL	0.80 [0.55; 1.24]***	0.72 [0.39; 1.21]***	0.55 [0.33; 0.93]	51.31; 0.0000*
Urea, mmol/L	9.03 [6.30; 12.70]	9.34 [6.08; 14.26]	9.78 [6.43; 13.94]	51.67; 0.0000*
Uric acid, µmol/L	68.71 [25.96; 119.96]	87.50 [32.11; 165.14]***	55.77 [22.18; 133.03]	14.55; 0.0022*
Lactic acid, mmol/L	1.72 [1.43; 1.95]	1.62 [1.41; 2.19]	1.66 [1.38; 2.03]	1.606; 0.6580
Pyruvic acid, µmol/L	13.85 [10.29; 19.36]	14.58 [10.29; 18.38]	13.73 [9.44; 19.49]	2.165; 0.5388
Albumin, mg/mL	0.311 [0.157; 0.590]	0.361 [0.217; 0.623]***	0.278 [0.177; 0.495]	5.448; 0.1418
α-Aminoacids, mmol/L	4.30 [3.91; 4.98]	4.23 [3.97; 4.98]	4.23 [3.88; 4.76]	31.40; 0.0000*
Imidazole compounds, mmol/L	0.315 [0.190; 0.451]	0.269 [0.190; 0.395]	0.273 [0.167; 0.402]	6.622; 0.0850**
Sialic acids, mmol/L	0.207 [0.159; 0.281]	0.192 [0.134; 0.262]	0.195 [0.140; 0.299]	2.760; 0.4301
Seromucoids, c.u.	0.100 [0.072; 0.154]	0.107 [0.073; 0.172]***	0.093 [0.055; 0.152]	4.904; 0.1790
ALT, U/L	3.85 [2.62; 5.30]	3.73 [2.62; 5.08]	3.92 [2.92; 5.15]	0.4223; 0.9356
AST, U/L	5.75 [4.00; 8.92]	5.92 [3.71; 7.75]	6.17 [4.25; 8.13]	2.594; 0.4585
AST/ALT-ratio, c.u.	1.51 [1.23; 1.84]	1.35 [1.15; 1.81]	1.48 [1.15; 1.90]	0.9801; 0.8061
LDH, U/L	1521.0 [885.3; 2044.0]	1357.0 [946.3; 2127.0]	1418.5 [761.4; 2022.0]	4.336; 0.2274
ALP, U/L	74.97 [47.81; 107.56]***	82.57 [54.33; 123.86]***	63.02 [41.29; 99.96]	18.36; 0.0004*
GGT, U/L	24.8 [19.3; 27.4]	23.4 [19.9; 26.0]	23.3 [20.2; 26.1]	10.81; 0.0128*
Catalase, nkat/mL	3.98 [2.00; 0.48]***	4.29 [2.85; 5.99]***	3.40 [2.44; 5.48]	26.38; 0.0000*
Superoxide dismutase, c.u.	81.6 [31.6; 139.5]	81.6 [34.2; 161.8]	67.1 [31.6; 142.1]	5.063; 0.1673
α-Amylase, U/L	413.3 [177.7; 798.0]	275.0 [101.1; 528.0]	304.9 [122.6; 579.0]	10.68; 0.0136*
Antioxidant activity, mmol/L	2.55 [1.40; 3.83]	2.29 [1.48; 2.94]	2.32 [1.49; 3.38]	1.476; 0.6879
Peroxidase, c.u.	0.335 [0.145; 0.730]	0.380 [0.230; 0.900]	0.430 [0.250; 0.830]	2.486; 0.4779
SOD/Catalase-ratio, c.u.	21.8 [5.6; 41.7]	21.9 [7.9; 37.3]	19.8 [9.2; 39.9]	9.953; 0.0190*
SOD/Peroxidase-ratio, c.u.	197.4 [90.8; 488.7]	139.8 [52.2; 423.3]	142.2 [65.8; 276.7]	3.404; 0.3334

Diene conjugates, c.u.	3.87 [3.69; 4.02]***	3.94 [3.73; 4.10]	3.92 [3.71; 4.18]	6.713; 0.0816**
Triene conjugates, c.u.	0.917 [0.831; 1.076]***	0.913 [0.785; 1.013]	0.865 [0.787; 1.010]	5.750; 0.1244
Schiff bases, c.u.	0.556 [0.494; 0.712]	0.552 [0.486; 0.664]	0.531 [0.476; 0.624]	5.387; 0.1456
MDA, μmol/L	6.84 [5.73; 11.11]	7.14 [5.77; 8.72]	7.05 [5.81; 8.55]	16.89; 0.0007*
SB/(DC+TC)-ratio, c.u.	0.114 [0.107; 0.143]***	0.111 [0.104; 0.133]	0.110 [0.099; 0.125]	11.28; 0.0103*
SB/TC-ratio, c.u.	0.592 [0.539; 0.695]	0.639 [0.560; 0.698]	0.620 [0.559; 0.680]	3.957; 0.2661
MM 254, c.u.	0.278 [0.182; 0.419]***	0.269 [0.167; 0.418]***	0.219 [0.163; 0.336]	5.643; 0.1303
MM 280, c.u.	0.214 [0.153; 0.348]	0.229 [0.145; 0.372]***	0.185 [0.130; 0.302]	5.194; 0.1581
MM 280/254	0.852 [0.756; 0.943]	0.867 [0.780; 0.943]	0.861 [0.792; 0.970]	2.797; 0.4240

Note. * - differences with the control group are statistically significant at p<0.05; ** - differences with the control group are statistically significant at p<0.10. Kruskal-Wallis test values are given to compare four groups: control group, ER (+), ER (++) and ER (+++). *** - Differences with ER (+++) are statistically significant, p<0.05.

Table S7. Biochemical composition of saliva in PR-positive and PR-negative breast cancer

Indicators	PR-negative, n=125	p-value PR-neg vs. Control	PR-positive, n=310	p-value PR-pos vs. Control	p-value PR-neg vs. PR-pos
pH	6.47 [6.20; 6.74]	0.5034	6.49 [6.24; 6.72]	0.8889	0.5826
Calcium, mmol/L	1.14 [0.84; 1.58]	0.0100*	1.27 [0.90; 1.64]	0.4996	0.0748**
Phosphorus, mmol/L	4.63 [3.44; 5.95]	0.3723	4.64 [3.54; 5.96]	0.1219	0.8559
Ca/P-ratio, c.u.	0.25 [0.18; 0.34]	0.0022*	0.27 [0.19; 0.37]	0.0448*	0.1325
Sodium, mmol/L	8.5 [5.4; 12.0]	0.9166	7.4 [4.6; 12.9]	0.0798**	0.2807
Potassium, mmol/L	11.6 [9.0; 14.7]	0.5519	11.0 [8.1; 14.2]	0.9485	0.6399
Na/K-ratio, c.u.	0.74 [0.49; 1.16]	0.6994	0.63 [0.47; 1.02]	0.0446*	0.2775
Chlorides, mmol/L	26.4 [21.6; 33.9]	0.2387	25.6 [19.8; 31.6]	0.8321	0.2495
Magnesium, mmol/L	0.298 [0.225; 0.382]	0.9436	0.294 [0.222; 0.375]	0.6987	0.8628
NO, μmol/L	29.3 [19.3; 43.7]	0.0241*	29.1 [16.9; 46.7]	0.0068*	0.8939
Protein, mg/mL	0.66 [0.36; 1.00]	0.0122*	0.61 [0.37; 1.08]	0.0047*	0.7741
Urea, mmol/L	9.53 [6.25; 13.10]	0.0000*	9.73 [6.25; 13.62]	0.0000*	0.8671
Uric acid, μmol/L	80.77 [26.92; 139.58]	0.1531	59.62 [22.28; 133.03]	0.0008*	0.3039
Lactic acid, mmol/L	1.67 [1.38; 1.97]	0.8962	1.63 [1.41; 2.07]	0.8873	0.8765
Pyruvic acid, μmol/L	14.46 [10.05; 19.36]	0.6612	13.97 [9.56; 19.12]	0.8389	0.7960
Albumin, mg/mL	0.286 [0.153; 0.553]	0.8193	0.311 [0.184; 0.556]	0.2175	0.4851
α-Aminoacids, mmol/L	4.24 [3.85; 4.81]	0.0001*	4.23 [3.91; 4.79]	0.0000*	0.4614
Imidazole compounds, mmol/L	0.277 [0.190; 0.379]	0.6074	0.281 [0.182; 0.410]	0.7974	0.7659
Sialic acids, mmol/L	0.207 [0.146; 0.281]	0.0741**	0.195 [0.140; 0.275]	0.1855	0.3356
Seromucoids, c.u.	0.095 [0.066; 0.163]	0.2755	0.099 [0.062; 0.152]	0.1232	0.9755
ALT, U/L	3.96 [2.88; 5.00]	0.5317	3.92 [2.77; 5.23]	0.6151	0.7853
AST, U/L	6.21 [4.42; 8.29]	0.0110*	6.00 [4.08; 8.08]	0.0960**	0.2229
AST/ALT-ratio, c.u.	1.56 [1.15; 2.05]	0.0407*	1.46 [1.14; 1.91]	0.2244	0.2234
LDH, U/L	1496.5 [887.7; 2057.0]	0.0059*	1438.0 [837.2; 2088.0]	0.0006*	0.9100
ALP, U/L	73.88 [47.81; 108.65]	0.0085*	71.71 [47.81; 102.13]	0.0786**	0.1954
GGT, U/L	22.9 [19.5; 26.6]	0.0002*	23.3 [20.2; 26.1]	0.0000*	0.5323
Catalase, nkat/mL	3.89 [2.48; 6.13]	0.0209*	3.65 [2.53; 5.83]	0.0001*	0.7078
Superoxide dismutase, c.u.	75.0 [39.5; 144.7]	0.0202*	71.1 [34.2; 140.8]	0.0222*	0.6613
α-Amylase, U/L	276.7 [111.6; 596.8]	0.0892**	320.8 [134.2; 611.4]	0.0001*	0.3566

Antioxidant activity, mmol/L	2.42 [1.43; 3.51]	0.7194	2.33 [1.49; 3.14]	0.2410	0.6279
Peroxidase, c.u.	0.470 [0.260; 0.880]	0.0964**	0.420 [0.250; 0.830]	0.1097	0.6692
SOD/Catalase-ratio, c.u.	22.7 [7.3; 37.2]	0.0041*	19.8 [8.4; 41.5]	0.0012*	0.6328
SOD/Peroxidase-ratio, c.u.	142.9 [47.2; 387.0]	0.5646	131.6 [65.8; 285.4]	0.4522	0.9507
Diene conjugates, c.u.	3.86 [3.70; 4.10]	0.0492*	3.93 [3.72; 4.13]	0.4993	0.2597
Triene conjugates, c.u.	0.898 [0.809; 0.990]	0.3512	0.893 [0.791; 1.020]	0.2028	0.9721
Schiff bases, c.u.	0.519 [0.476; 0.650]	0.8538	0.543 [0.485; 0.653]	0.0214*	0.3872
MDA, µmol/L	7.18 [5.81; 9.91]	0.0172*	7.09 [5.90; 8.80]	0.0118*	0.4978
SB/(DC+TC)-ratio, c.u.	0.110 [0.099; 0.129]	0.9787	0.111 [0.102; 0.128]	0.0924**	0.4221
SB/TC-ratio, c.u.	0.613 [0.553; 0.691]	0.2206	0.625 [0.561; 0.694]	0.0432*	0.9513
MM 254, c.u.	0.263 [0.164; 0.422]	0.8145	0.245 [0.167; 0.366]	0.2299	0.3695
MM 280, c.u.	0.216 [0.143; 0.371]	0.7546	0.198 [0.139; 0.322]	0.2963	0.3032
MM 280/254	0.869 [0.782; 0.954]	0.2447	0.856 [0.780; 0.962]	0.1669	0.7600

Note. * - differences are statistically significant at $p<0.05$; ** - differences are statistically significant at $p<0.10$.

Table S8. Biochemical composition of saliva in PR-positive (+, ++, +++) breast cancer

**Indicators**	**PR (+), n=64**	**PR (++), n=79**	**PR (+++), n=167**	**Kruskal-Wallis test (H, p)**
pH	6.50 [6.29; 6.73]	6.48 [6.27; 6.72]	6.48 [6.22; 6.71]	0.3261; 0.9551
Calcium, mmol/L	1.27 [0.98; 1.53]	1.37 [1.01; 1.86]****	1.25 [0.82; 1.57]	3.436; 0.3292
Phosphorus, mmol/L	4.41 [3.80; 5.98]	4.79 [3.55; 6.45]	4.65 [3.41; 5.74]	3.672; 0.2991
Ca/P-ratio, c.u.	0.27 [0.20; 0.36]	0.27 [0.20; 0.36]	0.27 [0.19; 0.37]	1.770; 0.6216
Sodium, mmol/L	6.6 [4.9; 12.6]	6.6 [4.5; 13.1]	7.9 [4.6; 12.9]	3.121; 0.3733
Potassium, mmol/L	11.3 [8.3; 14.1]	11.0 [8.1; 15.4]	11.0 [8.0; 14.1]	0.7834; 0.8534
Na/K-ratio, c.u.	0.62 [0.46; 1.14]	0.59 [0.43; 0.94]	0.73 [0.49; 1.03]	6.448; 0.0918**
Chlorides, mmol/L	26.9 [21.5; 33.0]	25.8 [20.6; 32.4]	24.9 [18.9; 31.2]	3.542; 0.3154
Magnesium, mmol/L	0.296 [0.233; 0.375]	0.292 [0.210; 0.371]	0.296 [0.232; 0.376]	0.5763; 0.9018
NO, µmol/L	26.5 [14.6; 43.3]	24.9 [12.5; 48.4]****	33.9 [20.9; 47.5]	17.72; 0.0005*
Protein, mg/mL	0.77 [0.51; 1.28]****	0.62 [0.31; 1.20]	0.57 [0.36; 0.98]	64.40; 0.0000*
Urea, mmol/L	9.98 [6.38; 13.64]	9.34 [6.43; 13.62]	9.65 [6.08; 13.68]	57.05; 0.0000*
Uric acid, µmol/L	74.46 [23.46; 136.04]	54.35 [22.94; 116.23]	56.49 [22.18; 143.56]	9.181; 0.0270*
Lactic acid, mmol/L	1.60 [1.27; 2.03]	1.67 [1.49; 2.24]	1.63 [1.36; 1.99]	3.951; 0.2668
Pyruvic acid, µmol/L	14.95 [10.29; 18.63]	14.58 [10.29; 21.32]	13.24 [8.82; 18.75]	3.751; 0.2896
Albumin, mg/mL	0.386 [0.243; 0.646]***, ****	0.305 [0.197; 0.513]	0.270 [0.165; 0.485]	12.82; 0.0050*
α-Aminoacids, mmol/L	4.33 [3.97; 4.78]	4.24 [3.90; 4.79]	4.20 [3.88; 4.84]	25.98; 0.0000*
Imidazole compounds, mmol/L	0.285 [0.193; 0.467]	0.285 [0.167; 0.395]	0.281 [0.190; 0.402]	3.181; 0.3646
Sialic acids, mmol/L	0.195 [0.146; 0.275]	0.201 [0.134; 0.278]	0.192 [0.134; 0.275]	0.3868; 0.9430
Seromucoids, c.u.	0.118 [0.088; 0.174] ***, ****	0.096 [0.064; 0.139]	0.094 [0.055; 0.158]	10.42; 0.0153*
ALT, U/L	4.08 [2.62; 5.08]	3.69 [2.46; 4.46]****	4.00 [2.92; 5.38]	3.716; 0.2938
AST, U/L	6.33 [4.50; 8.58]	6.00 [4.08; 8.08]	5.92 [4.08; 7.92]	1.678; 0.6417
AST/ALT-ratio, c.u.	1.59 [1.22; 2.09]****	1.61 [1.13; 2.15]****	1.36 [1.13; 1.77]	6.994; 0.0721**
LDH, U/L	1617.5 [826.8; 2082.5]	1457.0 [899.7; 2088.0]	1419.0 [809.9; 2088.0]	10.29; 0.0162*
ALP, U/L	84.75 [53.24; 126.03]***,****	69.54 [39.11; 99.96]	66.28 [45.63; 99.96]	12.87; 0.0049*
GGT, U/L	24.9 [20.9; 28.2]****	23.3 [21.0; 25.8]	22.6 [19.4; 25.7]	39.51; 0.0000*
Catalase, nkat/mL	4.74 [2.81; 6.56] ***, ****	3.36 [2.34; 5.29]	3.46 [2.47; 5.39]	19.46; 0.0002*

Superoxide dismutase, c.u.	78.9 [27.6; 140.8]	72.4 [47.4; 159.2]	65.8 [32.9; 139.5]	4.657; 0.1987
α-Amylase, U/L	551.0 [256.8; 798.0]****	335.1 [116.5; 568.0]	285.3 [122.6; 528.0]	18.57; 0.0003*
Antioxidant activity, mmol/L	2.64 [1.63; 3.38]	2.52 [1.61; 3.35]	2.17 [1.42; 2.98]	2.994; 0.3926
Peroxidase, c.u.	0.550 [0.290; 0.750]***	0.290 [0.210; 0.510]****	0.490 [0.250; 0.930]	5.971; 0.1130
SOD/Catalase-ratio, c.u.	15.5 [4.9; 40.8]***	23.3 [10.9; 48.8]	19.8 [8.2; 35.7]	8.615; 0.0349*
SOD/Peroxidase-ratio, c.u.	103.7 [52.6; 150.4]***	237.0 [137.8; 550.2]****	126.5 [57.6; 231.7]	12.30; 0.0064*
Diene conjugates, c.u.	3.94 [3.69; 4.10]	3.93 [3.71; 4.12]	3.93 [3.74; 4.17]	0.6841; 0.8769
Triene conjugates, c.u.	0.869 [0.796; 1.010]	0.853 [0.777; 1.013]	0.904 [0.810; 1.030]	2.646; 0.4495
Schiff bases, c.u.	0.521 [0.478; 0.623]	0.534 [0.477; 0.634]	0.557 [0.493; 0.678]	4.014; 0.2599
MDA, µmol/L	7.22 [6.15; 9.83]	7.22 [6.15; 9.83]	7.01 [5.73; 8.46]	15.74; 0.0013*
SB/(DC+TC)-ratio, c.u.	0.109 [0.103; 0.126]	0.110 [0.100; 0.128]	0.113 [0.103; 0.134]	2.464; 0.4818
SB/TC-ratio, c.u.	0.593 [0.548; 0.695]	0.629 [0.571; 0.684]	0.627 [0.561; 0.704]	3.053; 0.3836
MM 254, c.u.	0.273 [0.179; 0.398]	0.261 [0.180; 0.372]	0.220 [0.145; 0.349]	6.741; 0.0806**
MM 280, c.u.	0.196 [0.147; 0.348]	0.218 [0.147; 0.360]	0.185 [0.125; 0.290]	5.498; 0.1388
MM 280/254	0.849 [0.764; 0.933]	0.859 [0.801; 0.974]	0.852 [0.779; 0.967]	3.601; 0.3079

Note. * - differences are statistically significant at p<0.05; ** - differences are statistically significant at p<0.10. Kruskal-Wallis test values are given to compare four groups: control group, ER (+), ER (++) and ER (+++). *** - Differences with PR (++) are statistically significant, p<0.05; **** - differences with PR (+++) are statistically significant, p<0.05.

Table S9. Biochemical composition of saliva in ER/PR-positive and ER/PR-negative breast cancer

**Indicators**	**ER-negative + PR-negative, n=67**	**ER-positive + PR-positive, n=301**	**Kruskal-Wallis test (H, p)**
pH	6.47 [6.22; 6.71]	6.50 [6.26; 6.72]	0.2444; 0.8850
Calcium, mmol/L	1.15 [0.67; 1.48]***, ****	1.28 [0.90; 1.64]	3.974; 0.1371
Phosphorus, mmol/L	4.33 [2.75; 6.14]	4.64 [3.54; 5.97]	1.848; 0.3969
Ca/P-ratio, c.u.	0.257 [0.186; 0.370]	0.273 [0.195; 0.369]	2.108; 0.3485
Sodium, mmol/L	8.17 [5.37; 12.51]	7.40 [4.55; 12.91]***	4.796; 0.0909**
Potassium, mmol/L	11.85 [9.11; 14.85]	11.04 [8.07; 14.20]	0.5164; 0.7724
Na/K-ratio, c.u.	0.706 [0.455; 1.113]	0.635 [0.466; 1.024]***	6.813; 0.0332*
Chlorides, mmol/L	26.42 [21.94; 33.25]	25.60 [19.78; 31.76]	1.135; 0.5668
Magnesium, mmol/L	0.282 [0.209; 0.398]	0.294 [0.222; 0.379]	0.2629; 0.8768
NO, µmol/L	30.09 [19.30; 41.75]***	29.12 [17.37; 46.84]***	21.86; 0.0000*
Protein, mg/mL	0.71 [0.36; 1.09]***	0.61 [0.37; 1.09]***	48.78; 0.0000*
Urea, mmol/L	9.53 [5.79; 12.71]***	9.65 [6.22; 13.68]***	63.67; 0.0000*
Uric acid, µmol/L	86.63 [23.81; 139.58]	62.50 [23.14; 136.04]***	13.58; 0.0011*
Lactic acid, mmol/L	1.67 [1.38; 2.14]	1.64 [1.38; 2.07]	1.591; 0.4514
Pyruvic acid, µmol/L	14.46 [11.03; 20.59]	13.97 [9.80; 19.12]	2.402; 0.3009
Albumin, mg/mL	0.286 [0.154; 0.553]	0.312 [0.184; 0.559]	0.2845; 0.8674
α-Aminoacids, mmol/L	4.27 [3.84; 4.86]***	4.23 [3.91; 4.82]***	33.19; 0.0000*
Imidazole compounds, mmol/L	0.300 [0.228; 0.379]	0.281 [0.186; 0.410]***	4.073; 0.1305
Sialic acids, mmol/L	0.204 [0.128; 0.281]	0.195 [0.140; 0.281]	1.273; 0.5293
Seromucoids, c.u.	0.096 [0.072; 0.152]	0.095 [0.061; 0.155]	1.263; 0.5318
ALT, U/L	4.08 [2.62; 5.38]	3.92 [2.77; 5.23]	0.3041; 0.8590
AST, U/L	6.50 [4.67; 8.50]	6.00 [4.08; 8.17]	1.302; 0.5216
AST/ALT-ratio, c.u.	1.68 [1.11; 2.12]	1.46 [1.15; 1.90]	1.517; 0.4683
LDH, U/L	1687.0 [1024.0; 2217.0]***	1427.0 [837.7; 2088.0]***	8.423; 0.0148*

ALP, U/L	82.57 [52.15; 119.52]***, ****	71.71 [46.72; 102.13]***	14.30; 0.0008*
GGT, U/L	22.4 [19.8; 26.5]	23.3 [20.1; 26.1]***	14.30; 0.0008*
Catalase, nkat/mL	3.82 [2.37; 6.37]***	3.66 [2.53; 5.83]***	26.82; 0.0000*
Superoxide dismutase, c.u.	72.37 [47.37; 147.37]	71.05 [34.21; 142.11]***	3.769; 0.1519
α-Amylase, U/L	260.2 [116.5; 622.2]	323.3 [134.2; 611.4]***	14.70; 0.0006*
Antioxidant activity, mmol/L	2.14 [1.52; 3.33]	2.32 [1.49; 3.14]	0.3081; 0.8572
Peroxidase, c.u.	0.575 [0.360; 1.120]***, ****	0.430 [0.250; 0.830]	4.843; 0.0888**
SOD/Catalase-ratio, c.u.	22.36 [7.32; 37.77]***	20.07 [8.38; 41.54]***	11.16; 0.0038*
SOD/Peroxidase-ratio, c.u.	105.96 [26.32; 204.68]	131.58 [66.92; 296.52]	3.504; 0.1734
Diene conjugates, c.u.	3.99 [3.79; 4.25]	3.93 [3.72; 4.13]	2.638; 0.2674
Triene conjugates, c.u.	0.902 [0.784; 1.000]	0.891 [0.793; 1.020]	1.909; 0.3849
Schiff bases, c.u.	0.530 [0.488; 0.735]	0.544 [0.486; 0.653]	0.9348; 0.6266
MDA, µmol/L	8.38 [6.15; 10.17]***, ****	7.09 [5.81; 8.72]***	21.40; 0.0000*
SB/(DC+TC)-ratio, c.u.	0.110 [0.099; 0.130]	0.111 [0.102; 0.129]	2.649; 0.2660
SB/TC-ratio, c.u.	0.639 [0.569; 0.732]***	0.627 [0.561; 0.694]	2.949; 0.2289
MM 254, c.u.	0.295 [0.177; 0.426]	0.247 [0.167; 0.364]	2.563; 0.2776
MM 280, c.u.	0.238 [0.157; 0.380]	0.201 [0.141; 0.322]	2.878; 0.2371
MM 280/254	0.861 [0.777; 0.952]	0.857 [0.780; 0.965]	0.2256; 0.8933

Note. Kruskal-Wallis test values are given to compare three groups: control group, ER/PR-positive and ER/PR-negative breast cancer. * - differences are statistically significant at p<0.05; ** - differences are statistically significant at p<0.10. *** - Differences with control group are statistically significant, p<0.05; **** - differences between ER/PR-positive and ER/PR-negative breast cancer are statistically significant, p<0.05.
